# X-ray waveguide optics at GINIX/P10 PETRA III: recent progress and future directions

**DOI:** 10.1107/S1600577525011567

**Published:** 2026-02-10

**Authors:** Tim Salditt, Paul Meyer, Leon Merten Lohse, Jens Lucht, Jakob Soltau, Neele Kozák, Mike Kanbach, Markus Osterhoff, Fabian Westermeier

**Affiliations:** a Institut für Röntgenphysik Georg-August-Universität Göttingen 37077Göttingen Germany; b Deutsches Elektronensynchrotron (DESY), Notkestrasse 85, 22607Hamburg, Germany; Brazilian Synchrotron Light Laboratory, Brazil

**Keywords:** X-ray optics, X-ray waveguides, holo-tomography, PETRA IV

## Abstract

Here we report on recent progress in X-ray waveguide optics for full-field coherent imaging at the Göttingen Instrument for Nano-Imaging with X-rays (GINIX), installed at the P10 coherence beamline of the PETRA III storage ring at DESY, Hamburg. We describe fabrication methods including new materials and the corresponding characterization in terms of transmission, exit intensity distribution, far-field intensity distribution and coherence properties. In addition to single channels, which are currently used for holographic imaging, we include results on tapered waveguides and off-axis waveguide interferometers. We also address optimization of waveguide optics with respect to the novel super-resolution holography method presented by Soltau *et al.* [*Optica* (2021), **8**, 818–823]. Finally, we discuss the further development of high-resolution nano-holography in view of the planned storage ring upgrade to PETRA IV.

## Abstract 2

X-ray waveguide optics enable spatial and coherence filtering of a nano-focused synchrotron beam and provide tailored illumination wavefronts for holographic imaging. Progress in fabrication, characterization and advanced waveguide interferometers can enhance holographic imaging.

## 1. Introduction

**Figure 1:**
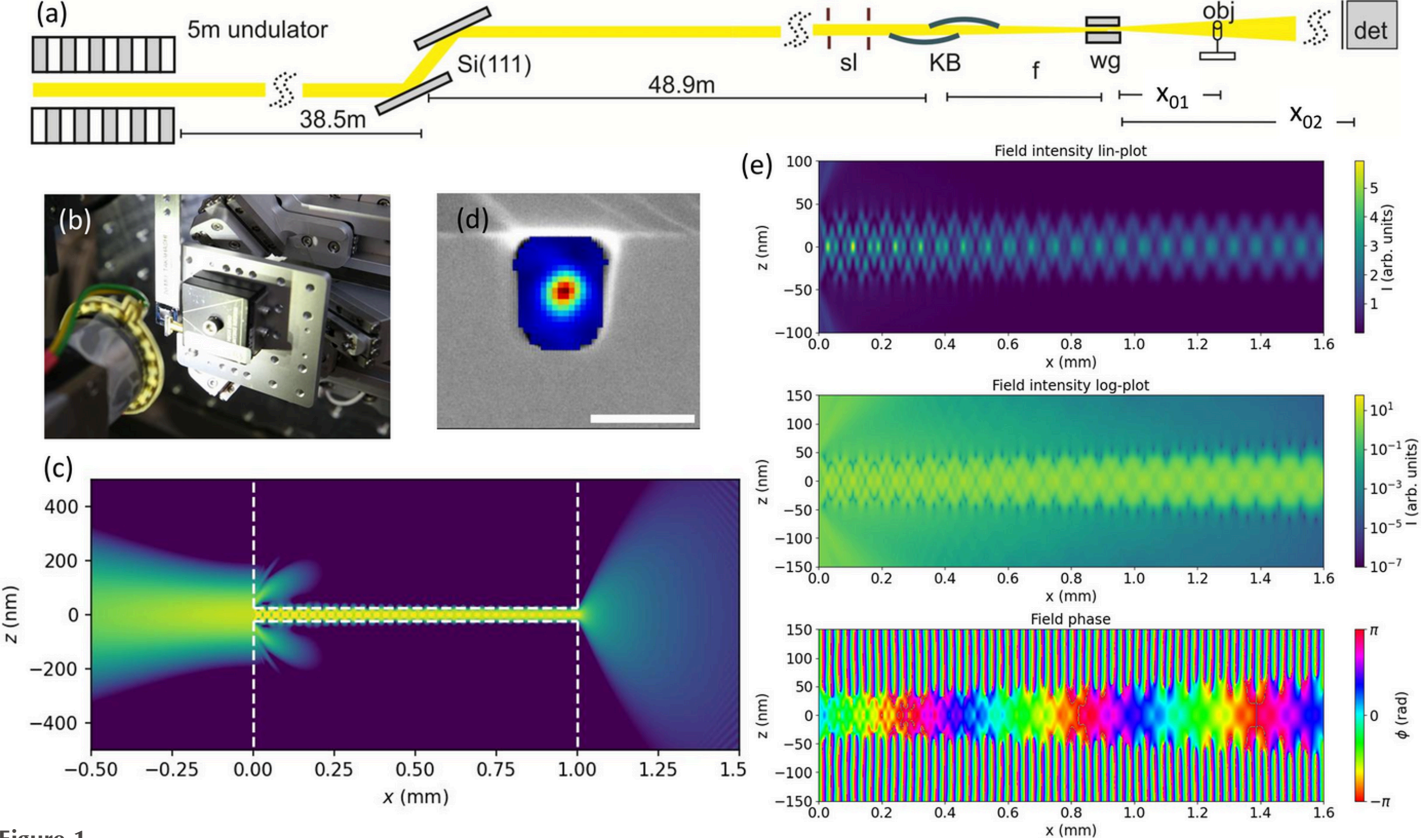
Illustration of beam path and WG optics for holographic imaging at the GINIX instrument. (*a*) Schematic of the beam path, starting with the 5 m undulator, the Si(111) monochromator and slit system. In the focus of the KB system, the WG is aligned for illumination of the object on the sample tower at defocus distance *x*_01_. The magnified hologram is recorded at distance *x*_02_ behind the WG with detectors mounted on the detector bench. (*b*) Photo of the WG stage, oriented against the beam. (*c*) FD simulation of front-coupling the KB (left) into the WG, propagation in the WG core (center), and WG exit field (right) for a (Si/air) WGb with *d* = 50 nm, *L* = 1 mm, at *E* = 8 keV. Comparing pre-focus of the KB (left) to WG exit (right), the increase in NA can be clearly noted. (*d*) SEM image of a WGb exit plane with superimposed reconstructed near-field intensity. This probe forms the quasi-point source for holographic imaging. Scale bar: 50 nm. (*e*) FD simulations of modal propagation in the WG, showing intensity in linear (top) and logarithmic (center) color scale, as well as phase (bottom) in a cyclic color scheme.

Consider the coherent illumination of an object positioned at a (defocus) distance *x*_01_ behind a synchrotron nano-focus, of which magnified wave-optical projection images are recorded in the detection plane at a distance *x*_02_ = *Mx*_01_ (see Fig. 1 ▸). For nanoscale imaging in this full-field scheme, the relatively large pixel size *px* ≃ 50–100 nm of current photon-counting detectors requires a large magnification *M* ≫ 1 in order to achieve effective pixel sizes *px*_eff_ = *px*/*M* in the range of ten to a few hundred nanometres. For indirect detectors, *M* can be relaxed by up to two orders of magnitude, but a typical geometry still inevitably implies very small Fresnel numbers 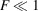, the so-called deeply holographic regime of propagation imaging. To illustrate this conclusion with typical numbers, let us assume a wavelength λ = 0.1 nm, a detector pixel size *px* = 10 µm and a desired sampling of *px*_eff_ = 20 nm over a field of view FOV = 40 µm (2*k* × 2*k* pixels). While the required magnification is given by the ratio *M* = *px*/*px*_eff_ = 500, the FOV and the numerical aperture NA of the nano-focusing optic (and thereby the illumination cone) determine the (minimal) defocus distance *x*_01_. Here, taking NA = 4 mrad as a typical value for a waveguide optic at this photon energy results in *x*_01_ = 10 mm. Therefore, the detector needs to be placed at distance *x*_02_ = *Mx*_01_ = 5 m from the focus and the (pixel) Fresnel number in fact becomes *F* = *px*^2^/(*M*λ*x*_12_) = 1 × 10^−4^, where *x*_12_ = *x*_02_ − *x*_01_ denotes the distance between object and detector, *i.e.* the wavefield propagation distance. In this regime of very small *F*, sensitivity to phase variations of the object exit phase is enhanced, and the phase problem is relatively well posed, compared with propagation imaging at larger *F* (Maretzke, 2018 ▸). High-performance phase retrieval techniques are now available (Maretzke & Hohage, 2020 ▸; Salditt & Robisch, 2020 ▸), no longer limited to linearization with respect to the object’s optical properties or with respect to the propagation distance (Huhn *et al.*, 2022 ▸; Lohse *et al.*, 2020 ▸; Lucht *et al.*, 2025 ▸). However, the coherence requirements are also very stringent in this regime, and high magnification is of course meaningless without high resolution. Therefore, the nano-focus optics and the secondary source used for illumination need to be carefully designed. The optic should in particular create a small spot size σ for high resolution and suppress background radiation which otherwise introduces errors in phase retrieval. In addition, it must also provide sufficient spatial and temporal coherence for the deeply holographic regime.

At the Göttingen Instrument for Nano-Imaging with X-rays (GINIX), installed at the P10 coherence beamline of the PETRA III storage ring at DESY in Hamburg, we have adopted a two-step approach to create a fully coherent and highly confined secondary source for holographic imaging. The compound nano-focus optical system is composed of a pair of high-gain fixed-curvature Kirkpatrick–Baez (KB) mirrors and an X-ray waveguide (WG) optic (Salditt *et al.*, 2015*b* ▸), which is positioned in the KB focal plane, for further spatial confinement of the beam (Krüger *et al.*, 2010 ▸; Bartels *et al.*, 2015 ▸) and for coherence filtering (Osterhoff & Salditt, 2011 ▸). By choice of the WG properties, notably the materials and width of its guiding core *d*, coherence, mode structure and wavefronts can be tailored. In other words, the WG system can be designed within certain limits to tailor the focus in shape, wavefront and coherence properties, and to suppress tails of the KB focal spot, based on attenuation of the radiative modes in the WG. In principle, WGs can be also used to ‘clean’ the focus of any other type of diffractive, refractive or reflective optical systems (Sakdinawat & Attwood, 2010 ▸; Schroer & Falkenberg, 2014 ▸), including Fresnel zone plate (FZP), compound refractive lens (CRL) or other types of focusing mirrors. However, since the limited WG transmission *T* < 1 also reduces the available photon flux for imaging, focusing systems of high efficiency such as KBs are desirable. Furthermore, the (single reflection) KB and WG compound optical system is achromatic, facilitating fast change of photon energy. Beyond serving as quasi-point sources for holographic imaging, WG optics also enable a variety of other functions, such as filtering, confining, guiding, coupling or splitting of beams (Salditt *et al.*, 2015*a* ▸; Hoffmann-Urlaub & Salditt, 2016 ▸).

In this work, we describe the set of X-ray WGs presently available at GINIX, also intended as a resource for users of the instrument, and report the recent progress in WG fabrication and characterization. This has now resulted in higher WG exit flux, reaching for example *I*_wg_ ≃ 3–5 × 10^9^ photons s^−1^ for a WG with a width of *d* = 80 nm. Beyond the increased transmission, a broader selection of WG materials, as well as different layouts for the WG channels are now available at GINIX. Apart from simple straight channels, we also present tapered channels and beam splitters for off-axis holography. Note that beam splitters have been reported before (Hoffmann-Urlaub & Salditt, 2016 ▸), but only up to small exit distances *D* = 3 µm, while we have now realized WG beam splitters up to *D* = 20 µm, more suitable for off-axis holography. Based on this progress in fabrication we can also present a first experiment of nano-holographic recordings in an off-axis interferometric setup: the beam is split into one beam that illuminates the sample, and one beam serving as an off-axis reference beam. Regarding the characterization of the WG optic, we include a gallery of far-field diffraction patterns, with corresponding probe reconstructions in the WG exit plane, and address the spatial coherence of the WG probe at GINIX based on speckle visibility. For optical design, we capitalize on progress in the optical theory of X-ray WGs (Lohse & Andrejić, 2024 ▸) and the associated simulation tools, which we use here to simulate modal properties and to optimize transmission, under the constraint of sufficient attenuation of the radiative modes. Finally, we address the question of which type of WG optics should be selected for super-resolution holography (SRH) (Soltau *et al.*, 2021 ▸), as well as future developments in view of the planned Coherence Application Beamline (CAB) at PETRA IV.

Fig. 1 ▸ illustrates the use of WG optics at the GINIX endstation of the PETRA III/P10 beamline. The instrumentation of the endstation, including upstream optics, slits, the KB, sample stage, online optical microscopes for alignment and inspection, flight path and detectors has been described in detail (Salditt *et al.*, 2015*b* ▸; Frohn *et al.*, 2020 ▸). Biomedical applications of holographic tomography (holo-tomography) with a WG probe have been reported (Töpperwien *et al.*, 2018 ▸; Reichardt *et al.*, 2020 ▸; Eckermann *et al.*, 2021 ▸; Frost *et al.*, 2023 ▸).

The basic optical scheme is sketched in Fig. 1 ▸(*a*): the monochromated undulator beam [Si(111) double crystal or channel-cut] is focused by a single-surface (Rh) fixed-curvature KB system, providing typical X-ray focal spot sizes of 250–350 nm (FWHM) in both directions with a flux larger than 10^11^ photons s^−1^ in a selectable energy range of 6–14 keV (Salditt *et al.*, 2015*b* ▸).

The KB beam is partially coherent, since it accepts a beam of 0.4 mm width, well above the horizontal coherence length. When closing the entrance slits to ∼50 µm, it becomes fully coherent, with a correspondingly diffraction broadened focal spot size (Giewekemeyer *et al.*, 2013 ▸). Alternatively, full coherence and reduction of the virtual source size for cone beam holography is achieved by inserting a WG optic in the focal plane of the KB. The function of the WG can hence be described as spatial and coherence filtering. Its exit plane forms a quasi-point source for coherent illumination and holographic imaging of an object positioned at variable defocus *x*_01_ behind the WG exit, as described above. The magnified hologram is recorded at *x*_02_ ≃ 5 m in the deep holographic regime, corresponding to small Fresnel numbers 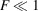. Two types of WG optics are available, denoted here as the bond-WG (WGb) and the crossed-WG (WGx) type. The WGx type is formed by two crossed sputter-coated planar thin film WGs (Fig. 4) with guiding layer made of C or BC_4_ in the thickness range 9 nm ≤ *d* ≤ 80 nm, and a two-component cladding consisting either of Ge/Mo or Ge/Ru, optimized for high transmission in the spectral range 11.1 keV ≤ *E* ≤ 20 keV. While the layer sequence Ge/Mo/C/Mo/Ge has been presented before (Salditt *et al.*, 2008 ▸; Krüger *et al.*, 2010 ▸; Krüger *et al.*, 2012 ▸), the Ge/Ru/BC_4_/Ru/Ge material system is new. Optically similar, the materials are chosen based on fabrication quality, notably concerning interfacial roughness and film stability. In contrast, the WGb type (Fig. 3) offers two-dimensional confinement not by crossing planar WGs, but is made of two-dimensional WG channels with air/vacuum as guiding layer (GL), and silicon as cladding layer (CL). The channels are fabricated by e-beam lithography, dry etching and wafer bonding (Hoffmann-Urlaub & Salditt, 2016 ▸). Improved lithographic fabrication has now resulted in a WG exit flux in the range *I*_wg_ ≃ 10^9^–10^10^ photons s^−1^, depending on the channel cross section. For alignment of the WGs, additional optical components (pinholes, cleanup apertures, wavefront modifiers) and an on-axis optical microscope are available. The object is positioned on the tomographic sample stage, under observation of a second on-axis optical microscope (Salditt *et al.*, 2015*b* ▸). The two microscopes are oriented parallel (pre-focus, WG alignment) and anti-parallel (post-focus, object alignment) to the beam.

Fig. 1 ▸(*a*) shows a schematic of the beamline layout and compound KB and WG optic. As a non-dispersive system, the photon energy can easily be scanned by coupled undulator and double-crystal monochromator scans. The WG positioning stage and the object microscope is depicted in (*b*), and the coupling scheme is illustrated by finite difference (FD) simulations in (*c*). The KB is focused onto the WG entrance, the exited modal field propagates over a distance of *L* = 1 mm along the empty channel in silicon. Radiative modes are attenuated, and the (filtered) WG beam is coupled out at the exit. The WG exit forms a secondary source size for holographic illumination. In (*d*), an overlay of the reconstructed intensity distribution and a scanning electron microscopy (SEM) image of the WG exit is shown, adapted from Soltau *et al.* (2021 ▸). Finally, (*e*) shows close-ups of simulated intensity and phase of the guided field within a channel in silicon.

In the next section, we will briefly recapitulate fundamentals of WG optics for holographic imaging, and then present simulations which can be carried out to predict the transmission and filtering properties of WGs for imaging at GINIX. This is followed by a section describing WG fabrication including novel material systems, and the corresponding characterization of WGs, highlighting the progress made with respect to earlier work, notably in usable exit flux; we also present an overview of available WGs. We then present progress in WG interferometers, created by split WG channels. The article closes with an outlook on WG optics for SRH and the PETRA IV upgrade project.

## 2. Design and simulation of X-ray WG optics for holographic imaging

The design of X-ray WG optics must take into account the parameters of the beamline, in particular the focusing optics and photon energy *E*, the spot size and NA for holographic illumination, and the available material and fabrication constraints. To this end, efficient numerical tools for wavefield propagation based on FD solvers of the parabolic wave equation (Fuhse & Salditt, 2006 ▸; Melchior & Salditt, 2017 ▸) are readily available on github (Lohse, 2025*a* ▸). The well established analytical treatment of guided waves provides the fundamentals. Monochromatic propagation of modal wavefields in a planar X-ray WG along the optical axis *x* can be written in scalar wave theory as a superposition of a discrete set of *N* guided modes,
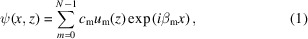
where *u*_m_(*z*) is the envelope of the mode along the orthogonal direction along which the WG is structured, parameterized by its refractive index profile *n*(*z*), and β_m_ is the propagation constant. The modes *u*_m_(*z*) are eigenfunction solutions of the reduced wave equation, 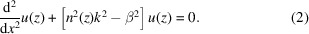
For simplicity, we note down only the one-dimensional case (planar WGs).

The number *N* of modal solutions rapidly decreases with the width of the potential down to the regime of mono-modal waveguiding. For a simple rectangular profile with guiding layer thickness *d*, *u*(*z*) is given by harmonic functions with *m* + 1 antinodes in the guiding layer and an exponentially decaying evanescent wave in the cladding (Marcuse, 1974 ▸). When *d* becomes equal to or smaller than the critical width *W*, only a single mode can propagate in a layer of index *n*_1_ and thickness *d*, in between a cladding of *n*_2_ (Bergemann *et al.*, 2003 ▸):
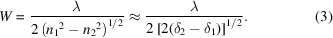
Evaluating *W* for X-ray wavelength and material constants *n* = 1 − δ with δ = *r*_0_ρλ^2^/2π, where *r*_0_ is the Thomson scattering length (classical electron radius) and ρ the electron density of the material, we obtain a fundamental length scale for X-ray WGs. For single-material WGs, formed by an air/vacuum guiding layer and a metal cladding, *W* can be expressed in terms of the critical angle θ_c_ and (away from absorption edges) by the material electron density ρ_e_:
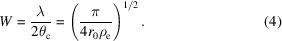
For literature density values, we obtain *W* = 20.0 nm for Si, *W* = 14.2 nm for Ge and *W* = 10.3 nm for Mo, independent of λ, as long as the scaling for δ holds (away from absorption edges). The modal coefficients *c*_m_ are calculated by an overlap integral of the incident field ψ_in_ impinging onto the front side of the WG and *u*_m_ (Bongaerts *et al.*, 2002 ▸; Fuhse & Salditt, 2006 ▸):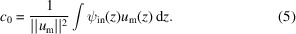 An ‘effective linear absorption coefficient’ μ_eff, m_, given by a mode-weighted average of the absorption coefficient profile μ(*z*) (Fuhse & Salditt, 2006 ▸), 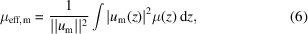
determines the damping of the mode 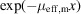, and is added in the right-hand side of equation (1). For a WG with an air or vacuum guiding core, only the intensity fraction in the cladding contributes to the absorption of the mode. The treatment of modes as an eigenvalue problem with a Hermitian operator as in equation (2) is approximative. It neglects radiative modes and assumes that the functions *u*_m_(*z*) depend only on the real-valued decrement δ(*z*) of the index of refraction *n*(*z*) = 1 − δ(*z*) − *i*β(*z*), but not on β(*z*). Absorption is introduced only *a posteriori* by equation (6). A more general and complete nano-optical theory of X-ray WGs based on computing the Green’s functions has recently been given (Lohse & Andrejić, 2024 ▸). Apart from cases of strong material absorption, this approach offers the advantage that the propagation constant, mode functions *u*(*z*) and the attenuation length *l* can be computed numerically for general piece-wise constant potentials. Further, evanescent coupling in grazing incidence, front coupling in forward incidence and radiation from buried emitters can be treated (Lohse *et al.*, 2025 ▸). We can now capitalize on this progress and use the resulting numerical package *XWG* (Lohse, 2025*b* ▸) to design WGs for holographic imaging.

**Figure 2:**
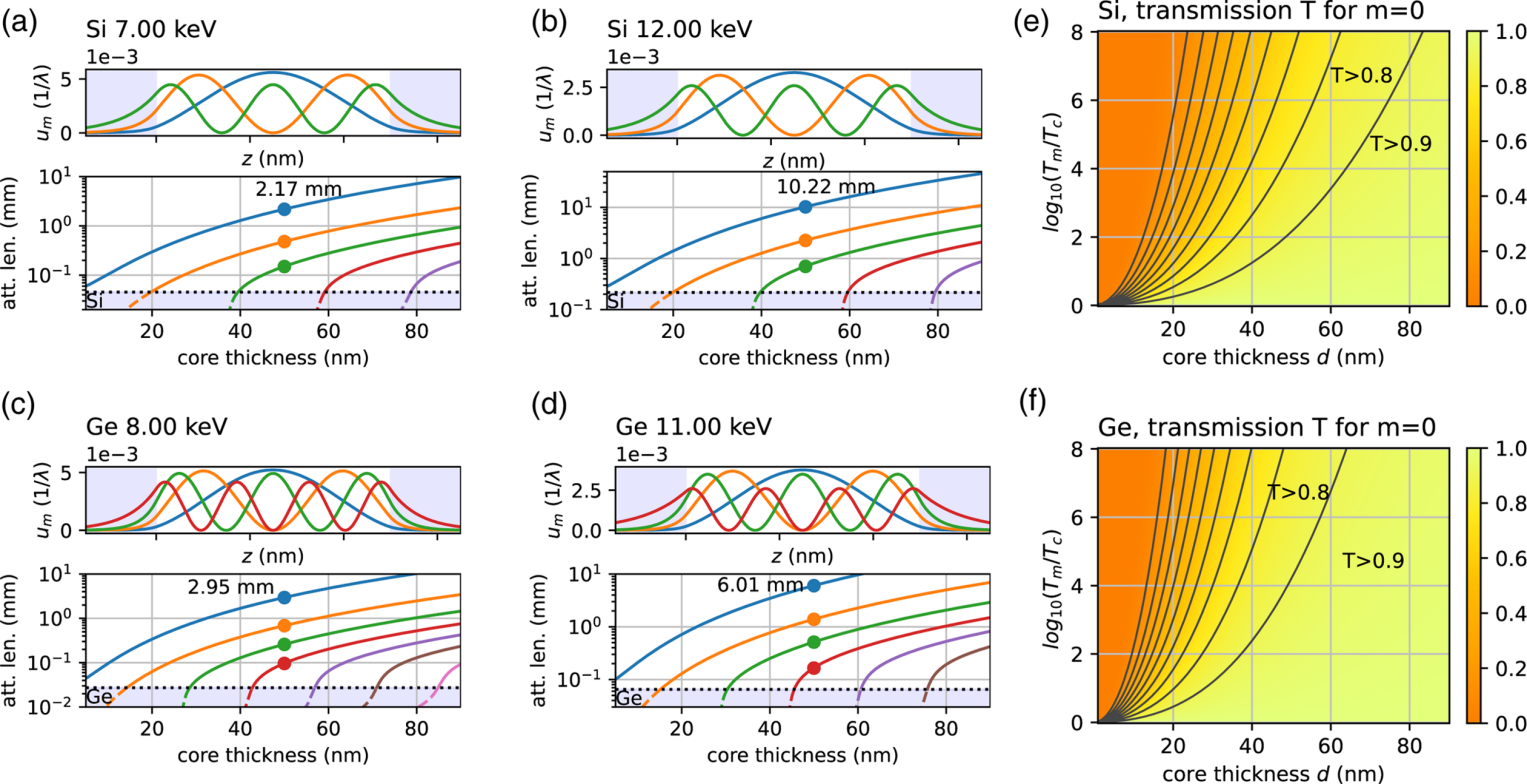
WG optical design: simulated modal functions |*u*_m_(*z*)|^2^, attenuation length *l*_m_ and achievable transmission *T*. (*a*–*d*) upper panel: mode intensity distribution for *d* = 50 nm; lower panel: attenuation length *l*_m_(*d*) as a function of *d*, for photon energy (*a*) *E* = 7 keV, (*b*) *E* = 12 keV for Si cladding, and (*c*) *E* = 8 keV, (*d*) *E* = 11 keV for Ge cladding. The different colors show the respective modes *m*. (*e*–*f*) Modal transmission *T*_0_ of the fundamental mode *m* = 0, as a function of *d* and *T*_m_/*T*_c_ = 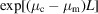 for (*e*) Si, (*f*) Ge. The colors (see color bar) encode the transmission *T*_0_.

Fig. 2 ▸ shows the mode functions |*u*_m_(*z*)|^2^ and the attenuation length *l*, computed by *XWG* as a function of the guiding core thickness *d*, for WGs made from Si and Ge as cladding materials. The guiding core is assumed to be air or vacuum, representing the WGb type of guides, fabricated by e-beam lithography and a subsequent wafer bonding process, which is well established for single-crystal wafers of these two semiconductors. Results are shown for each material at two photon energies, namely (*a*) *E* = 7 keV and (*b*) *E* = 12 keV for Si, and (*c*) *E* = 8 keV and (*d*) *E* = 11 keV for Ge. These values represent the range of *E* where WGb-type optics can be used at GINIX, while higher *E* can be covered by materials of the WGx type. For each of the four cases (*a*–*d*), the upper panel shows the modal intensity distributions |*u*_m_(*z*)|^2^ in a guide of fixed thickness *d* = 50 nm. For this value, Si supports three modes (*m* = 0, 1, 2), while Ge (as the ‘deeper potential well’) already has four modes – see the profiles of different colors. The modes exhibit the expected symmetric shape with *m* zeros, and the exponential tail in the cladding. The only contribution to absorption in this WGb type is by the tails of the modes propagating in the cladding, while the guiding core with air/vacuum does not contribute. Since the fraction propagating in the cladding increases with *m*, the corresponding modal attenuation length *l*_m_ decreases. This is shown in the lower panels Figs. 2 ▸(*a*)–2 ▸(*d*) as a function of *d* – see the different colored curves representing *l*_m_(*d*). Starting from the value of the cladding *l*_m_ = *l*_c_ when the thickness just reaches the critical values *d* = *d*_m_, where an additional mode *m* is supported, the attenuation length rapidly increases to *l*_m_ ≫ *l*_c_, when the next mode is reached at *d* = *d*_*m*+1_ (note the semi-logarithmic scaling). The corresponding transmission *T*_m_ of the mode for given WG length *L* hence rapidly exceeds the transmission of the cladding *T*_c_. Of course, both *T*_c_ and *T*_m_ decrease with *L*, the first effect wanted, the second not.

Next, we address the modal transmission *T*_m_ as a function of *d* and the ratio *T*_m_/*T*_c_. The design parameter *d* directly affects resolution, while *T*_m_/*T*_c_ is the intensity ratio of the WG beam to the transmitted radiative modes (direct KB beam attenuated by the cladding), or equivalently the WG signal-to-background ratio in the exit plane of the WG.

Both are chosen in view of the experimental needs. We are now primarily interested in the fundamental mode *m* = 0, since for a rectangular potential and plane wave illumination it carries the largest fraction 8/[(*m* + 1)π^2^] ≃ 0.81 of the intensity. For a single-material WG, the modal transmission *T*_m_ at fixed ratio *T*_m_/*T*_c_ depends on *d* but is independent of *E*. This is because the ratios of the respective attenuation lengths is given by the intensity fraction of the mode propagating in the cladding (cladding intensity fraction), which is independent of *E*. The WG length *L* does not enter, because it is set by the ratio *T*_m_/*T*_c_ = 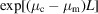, where μ_c_ and μ_m_ denote the absorption coefficients of cladding and mode, respectively. The resulting figure of merit *T*_m_ is hence characteristic only for the material and *d*, the only quantities that determine the cladding intensity fraction. Figs. 2 ▸(*e*) and 2 ▸(*f*) show the results for Si and Ge, respectively. We see that for *d* ≃ 50 nm and*T*_c_/*T*_m_ ≃ 0.0001, we obtain *T* ≃ 0.9 for Ge and *T* ≃ 0.8 for Si, which are both quite tolerable losses. In fact, most of the intensity of the KB focus is lost not by attenuation of the mode, but by the coupling efficiency. This is primarily due to the geometric mismatch, when the focal spot of the pre-focus optic is much larger than *d*, a situation that can be expected to change at PETRA IV, based on better focusability of the high-brilliance beam.

Finally, a brief note to explain why we presented the photon energies shown. *E* = 7 keV is the lowest value at which GINIX is operated, and below *E* = 8 keV, the absorption becomes too high in Ge, when assuming a minimum WG length 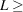 0.20 mm which can still be diced. At the other end, we take a length limit into account for the lithographic fabrication *L* ≤ 2 mm, limiting the photon energies to about *E* ≤ 12 keV. For higher *E*, Si becomes too transparent, *i.e.* the radiative modes are not sufficiently damped. For Ge, on the other hand, we do not want to operate the WG directly above the *K* edge, because otherwise the absorbed dose becomes high and a photo-induced surface reaction possibly associated with an oxide layer is observed. Note that for higher *E*, the WGx-type optic is recommended.

## 3. WG types, fabrication methods and materials

At multi-keV photon energy *E* and targeted cross sections *d* ≤ 100 nm, X-ray WGs are structures of high aspect ratio (length *L* to width *d*), easily reaching the range of *L*/*d* ∈ [10^4^, 10^6^]. These values along with the required small interfacial roughness impose significant challenges in fabrication. The first WG channels supporting two-dimensional beam confinement were made of stripes of negative photoresist acting as guiding layer patterned by e-beam lithography. These stripes were then coated with metal or a semiconductor cladding layer (Pfeiffer *et al.*, 2002 ▸; Jarre *et al.*, 2005 ▸). Since photoresist is unstable at high X-ray dose, this was later replaced by dry etching (RIE process) of channels into silicon wafers and subsequent capping by wafer bonding (Neubauer *et al.*, 2014 ▸; Hoffmann-Urlaub & Salditt, 2016 ▸; Bartels *et al.*, 2015 ▸). Denoted as WGb (‘bond’ WG), the resulting WGs are single-material devices. The channels forming the guiding layer are filled with air or vacuum. Since the WGb scheme relies on wafer bonding, it is restricted to semiconductor single-crystal materials, notably Si, Ge and GaAs.

**Figure 3:**
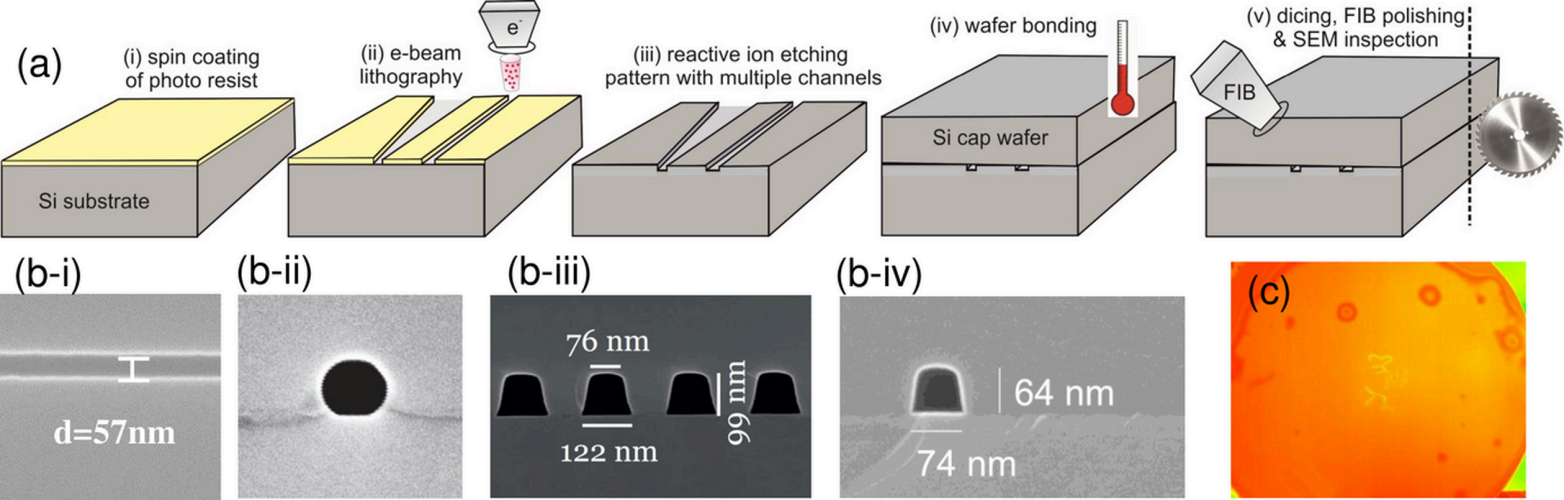
Fabrication of bonded WGs (WGb) based on e-beam lithography and wafer bonding. (*a*) Principal processing steps: spin-coating of PMMA photoresist, e-beam lithography, dry etching, wafer bonding, dicing. (*b*) SEM inspection of (*b*-i) an etched channel in Si, (*b*-ii) exit face of a Ge channel, (*b*-iii) WG array in Si, and isolated channel in Si, with indicated width and height. (*c*) Infrared image of bonded wafer, showing some air inclusions.

Fig. 3 ▸ gives an overview of WGb fabrication, with (*a*) schematics of fabrication steps, (*b*) SEM images of different channels in Si and Ge, and (*c*) inspection of a bonded wafer by infrared microscopy. The WGb-type lithographic fabrication also allows for more advanced schemes of tapered and split channels, as required for off-axis holography. In (*b*-i) a SEM image of an etched Si channel is shown, recorded in top view before bonding, in (*b*-ii) a Ge channel is depicted in front view. The Ge WGs represent a new development at the Institute for X-ray Physics (IRP). Surface diffusion during the bonding process results in very smooth and round cross sections, but due to material processes in the Ge oxide these channels deteriorate in X-ray beams of high intensity, at least when kept in air (see the inspection report before and after intense irradiation, included as supporting information). Fabrication of Si WGb at IRP follows the protocols described by Hoffmann-Urlaub & Salditt (2016 ▸). Based on the instrumentation and processes used, the channel quality is satisfactory down to channel widths of *w* ≃ 100 nm. Enhanced quality in particular also for small *w* is offered commercially by Eulitha AG (Würenlos, Switzerland), but only for write fields up to 2 mm × 2 mm and for Si. Typically, the channel quality is satisfactory down to channel width of *w* ≃ 50 nm. The higher interface quality (lower roughness) has resulted in a transmission gain in recent years compared with previously published protocols implemented completely in the clean room at IRP (Neubauer *et al.*, 2014 ▸; Hoffmann-Urlaub & Salditt, 2016 ▸; Bartels *et al.*, 2015 ▸). In practice, this allows one to reach a WG exit flux on the order of 10^9^ photons s^−1^ also for sub-30 nm beam confinement, with a correspondingly high divergence of the exit beam. Unfortunately, it is very difficult if not impossible to quantify roughness inside the channels of WGb type. While SEM gives a visual impression and allows one to assess larger defects and imperfections such as waviness, microscopic roughness of the channel walls and interfaces is elusive. More importantly, after bonding, the interior interfaces change due to atomic mobility at the elevated temperatures. For visual comparison, SEM images of old and new WGb channels are presented side by side in the supporting information. Fabrication via e-beam lithography (EBL) of larger write fields with longer channels as well as other semiconductor materials (Ge, GaAs) is done at IRP, since it is not available commercially. Here, channel length up to *L* ≃ 5 mm is enabled by the laser interferometric fixed-beam moving stage of the *eLine* EBL tool (Raith GmbH, Dortmund, Germany). Wafer bonding is always carried out at IRP, following the protocols detailed by Hoffmann-Urlaub & Salditt (2016 ▸), Hoffmann-Urlaub (2017 ▸). In an ongoing development, this scheme has now been extended to Ge, see Fig. 3 ▸(*b*-ii), with adapted gasses/values for the RIE and the bond process, As visible in the micrograph, the high surface mobility during the bonding process results in a cylindrical channel diameter. While this thermal process may go along with [be accompanied by smoothing, which in addition to the higher electron density of Ge is beneficial for imaging (see the section below), in particular at energies just below the Ge *K* edge at *E* = 11.103 keV, we found the Ge channels to be unstable after longer exposures to the focused synchrotron beam in ambient conditions (humidity, temperature), possibly due to the formation of an oxide or oxide-related degradation processes. While intact, the tested Ge channels gave very satisfactory transmission and far-field pattern (see the next section). An extension to WGb made of GaAs is envisioned, with preliminary wafer bonding results already achieved (Kozák, 2021 ▸).

The current WGb collection available for user experiments at GINIX is fabricated on silicon wafers of the following type: P-Si (boron) 〈100〉, 100.0 (5) mm diameter, 0.525 (25) mm thickness. After standard RCA cleaning of the Si wafer surface, the wafer is coated with a 140 nm-thick layer of poly(methyl methacrylate) (PMMA) on the Si surface and exposed with an EBL tool for e-beam lithography (Vistec EBPG5000) at 100 keV electron energy. A dose array of ten doses ranging from 500 µC cm^−2^ to 2200 µC cm^−2^ with 18% steps is applied, for later inspection and choice of optimum results. Following the exposure, the structure is developed within a solution made of 3:1 isopropyl alcohol and MIBK for 45 s. Anisotropic Si etching is then performed on the Oxford Plasma lab system 100, using a mixture of gasses SF_6_ and C_4_F_8_. After reaching the desired etching depth, the remaining PMMA resist is removed with oxygen plasma. All of the above steps are carried out by Eulitha AG (Würenlos, Switzerland). The arrays of channels are grouped in eight write fields of 2 mm × 2 mm size exposed with different electron doses. At IRP, the obtained silicon molds are then inspected by SEM and the write fields offering the best quality are identified. The entire wafer is then capped by wafer bonding. For bonding of Si, the wafers are placed on top of each other by hand, gently pressed and annealed in an oven, heated to *T* = 1000°C at a rate of 4°C min^−1^ and then kept at this temperature for 4 h. For Ge the ramping rate is 3°C min^−1^ and the plateau bonding temperature is *T* = 600°C for 5 h. In both cases, the oven is flushed with N_2_ at 50 l h^−1^. The bonding quality is inspected by an infrared microscope, in which air inclusions and unbonded areas can be detected [see Fig. 3 ▸(*c*)]. Finally, the WG pieces corresponding to the selected write fields are diced out to the desired length *L*. Since the dicing would obstruct the channels, the wafer is diced from both sides, but only up to a thin slice containing the bond interface. This part is then gently broken. The resulting channel entrances and exit faces are inspected by SEM [see Fig. 3 ▸(*b*-ii,-iii,-iv)]. The available collection of WGb types with different collections of WG channels is used at GINIX at lower photon energies 7 keV ≤ *E* ≤ 10 keV, while materials other than Si and larger write fields are not yet available in sufficient quality and quantity. They offer beam confinement in the range 20–60 nm (FWHM) and sufficient transmission *T*.

**Figure 4:**
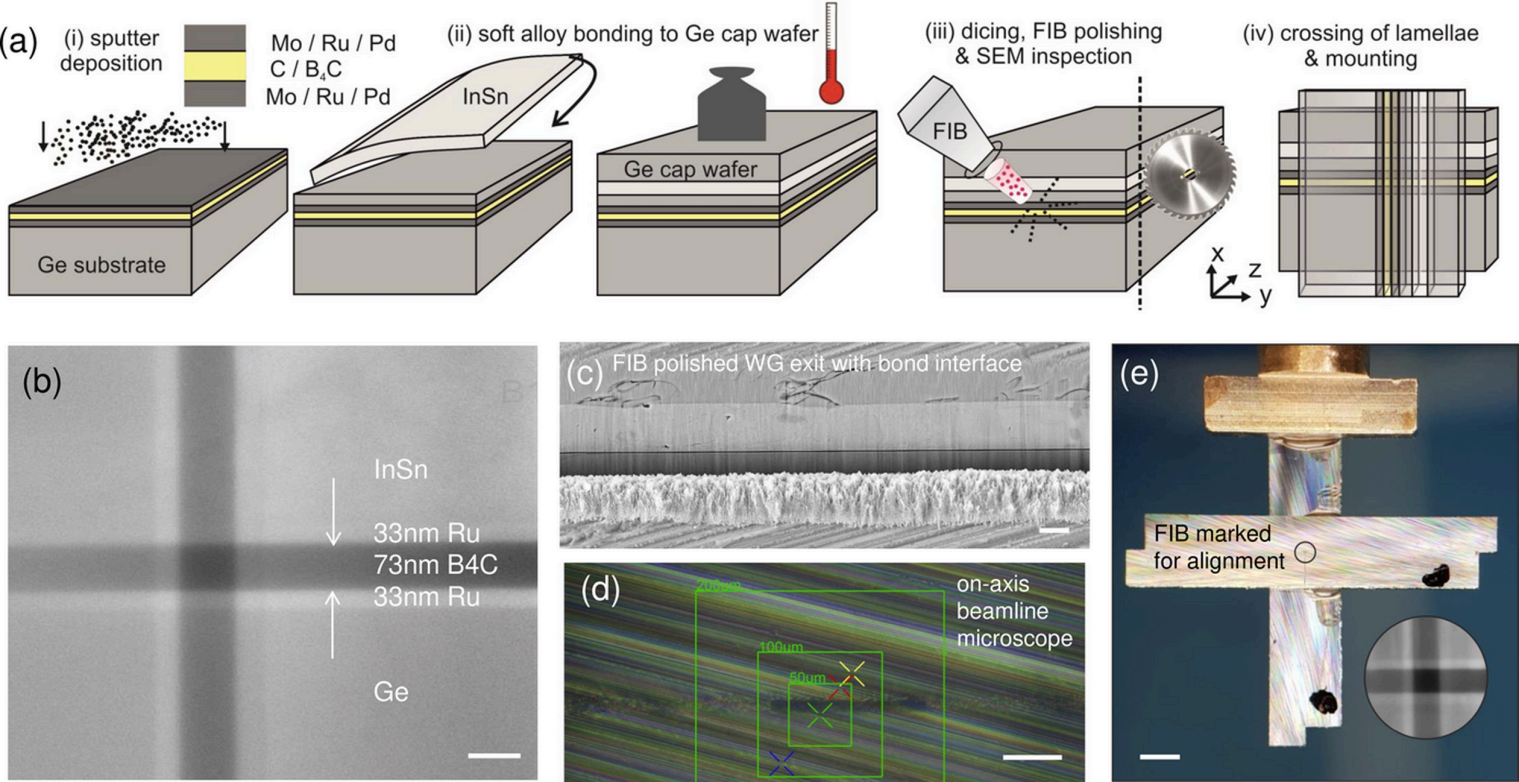
Fabrication of crossed planar WGs (WGx type) by thin film deposition, soft alloy bonding and crossing of two planar WG lamellae. (*a*) Principal processing steps: sputter deposition of the desired layer sequence, soft alloy bonding, dicing, FIB polishing, crossing of two lamellae to form the WGx-type WG. (*b*) Overlay of two SEM images of a Ru/B_4_C/Ru WG, with indicated layer width. (*c*) SEM image showing the bond interface after FIB milling. (*d*) WGx alignment in the optical microscope at GINIX. The horizontal dark stripe corresponds to the region treated by the FIB. The diagonal lines are traces of the dicing in the Ge wafer. (*e*) Crossed lamellae forming the WGx, mounted on a holder, and ready to be used at the beamline.

Fig. 4 ▸ shows an alternative fabrication scheme for 2DWGs based on two planar WGs (1DWG), which are crossed and which each confine the beam in one of two orthogonal directions. Combined in a crossed geometry, they form the WGx-type WG optic creating a two-dimensional source for holographic imaging (Krüger *et al.*, 2010 ▸). In other words, an effective two-dimensionally confining WG (2DWG) source is simply obtained by crossing of two planar (one-dimensionally confining) WGs. Without the need to define channels by lithography, fabrication of the 1DWGs is hence compatible with thin film deposition techniques. This also opens up the possibility of designing a plethora of possible thin film sequences, including WG arrays (Zhong *et al.*, 2017*b* ▸). Small guiding layers down to the fundamental limit of beam confinement (Bergemann *et al.*, 2003 ▸) can also be realized. Fabrication challenges are rather associated with larger film thicknesses which can create stress. The deposited layer sequence has also to be capped with a second substrate wafer to block radiative modes. This is achieved via soft alloy bonding (Krüger *et al.*, 2010 ▸). In summary, smaller guiding layers, a wider range of materials and more complex layer sequences can be realized, including two-component claddings optimized for high transmission (Salditt *et al.*, 2008 ▸). Using for example an interlayer made of Mo, placed between the guiding core (C) and a high-absorption cladding (Ge), this scheme provides excellent WGs for the photon energy range between the Ge *L* edges and the Mo *K* edge. As downsides, the material of the guiding layer, even though of low electron density, also contributes to absorption of the guided modes, in particular at lower photon energy, and splitting or tapering of WGs is not possible. The collection of WGx-type WGs has now been extended with two more materials, notably a Ru/B_4_C/Ru and a Pd/B_4_C/Pd system. Note that material combinations are chosen not only in view of a suitable index profile with a high difference of electron density between guiding core and cladding, but also in view of their compatibility with thin film deposition techniques with high interfacial quality. For example, in terms of density and optical constants, the guiding core materials C and B_4_C may be very similar, but for a given metal and sputter deposition instrument and protocol, the interfacial roughness σ may be very different. In development of the protocols, interfacial roughness was measured by X-ray reflectivity and found to be in an acceptable range σ ≤ 0.5 nm. The magnetron sputtering of the new planar WG systems was carried out at DESY, using the instrumentation and protocols described by Lohse (2024 ▸). Far-field patterns and typical intensity values are presented in the next section.

## 4. WG characterization

Following design and fabrication, the next step is characterization of the WGs. This requires alignment of the WG in the KB beam at GINIX, but in fact the KB itself is first aligned using a known WG optic.

**Figure 5:**
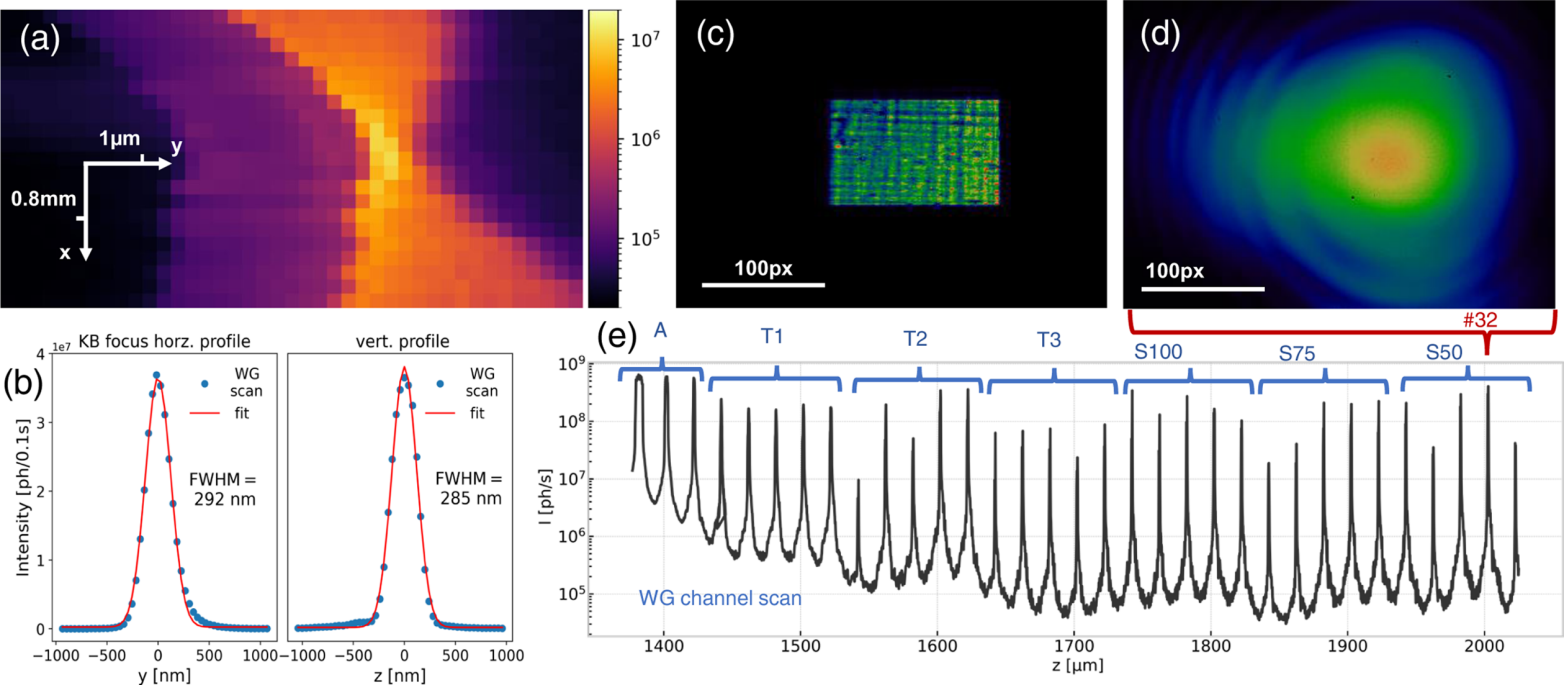
WG alignment in the KB beam. (*a*) Intensity distribution of the KB focal region, probed at *E* = 13.8 keV, by scanning *WGx-Ru80-300* (*d* = 80 nm, *L* = 300 µm, Ru/B_4_C/Ru) through the focal region. This can be recorded for different angles of the KB mirrors to align the KB and to find the focal plane. (*b*) The final KB focal width is controlled by scanning a WGx through the focal plane, after identification of the focal plane along the optical axis. The integrated intensity profiles (Pilatus 300k detector) in the two orthogonal directions along with least-square fits, shown for *WGx-CH* (*d* = 80 nm, Mo/C/Mo) at *E* = 13.8 keV. Focal widths around FWHM = 300 nm are typical (independent of *E*). (*c*) Far-field intensity pattern of the KB, measured with attenuators. (*d*) Far-field intensity pattern of *WGb-Ev3R3* channel #32 at *E* = 8 keV, recorded with the Eiger detector (linear color map, 0–2.8 × 10^4^ photons). (*e*) Scan along the bond interface of *WGb-Ev3R3* through the KB focus at 8 keV. The integrated intensity of the Pilatus 300k detector shows the maxima corresponding to channels #4–#32, consisting of broad channels for alignment (A), tapered channels (T1–T3) of different taper angles and straight channels (S) of different nominal width *w* = 100, 75, 50 nm.

Fig. 5 ▸ illustrates the general procedure. In (*a*), the focal field distribution of the KB is shown in the *xy* plane (orthogonal to the optical axis), obtained by measuring the transmitted intensity during a lateral scan of the WG, after finding the optimal angles of incidence on the KB mirrors, essentially by rerunning the scan in (*a*) for different KB settings, given by the positions and rotations of the mirrors in the *x* and *y* directions. In (*b*) the final scans along the principal axis perpendicular to the optical axis indicate the spot size that has been achieved. Typical values for the FWHM are around 280–350 nm. They depend on the electron orbit of the storage ring, overall beamline alignment and the WGs used. The profiles correspond to the intrinsic KB spot size convolved with the WG acceptance. Note that the KB alignment cannot be inferred from the far-field pattern alone, which is shown in (*c*).

The rectangular shapes reflect the divergence and also reveal the partially coherent nature. The structure of the beam results from imperfection of the mirrors, and here also from the attenuators that were used in order to record the KB on the Eiger4M pixel detector (Dectris). In contrast to the KB, the far-field pattern of the transmitted waveguided beam is smooth and fully coherent – see panel (*d*). In the case of WGb, the desired WG channel can be chosen from the multiple channels, by scanning along the interface – see (*e*) for the example of *WGb-Ev3R3*.

From the data such as in Figs. 5 ▸(*c*), 5 ▸(*d*) the WG transmission can be calculated for a given alignment setting and photon energy. An example of typical values at which holographic experiments are carried out with the WGb-type optics is the following: for *WGb-E6B2* with a channel cross section of 109 nm × 112 nm (measured by SEM) and optical depth *L* = 1.2 mm, an integrated far-field of *I*_0_ = 1.76 × 10^9^ photons s^−1^ is measured at 8 keV. Moving the WG out of the beam, the KB beam impinging on the detector with higher flux and with smaller NA has to be attenuated. Correcting for the attenuation, the KB beam intensity is determined to *I*_0_ = 6.6 × 10^10^ photons s^−1^. At the same time, the KB spot size is measured to 334 nm × 272 nm (FWHM), by inserting the WG again and scanning it through the beam. Correcting for the over-illumination, *i.e.* mismatch in KB spot size and channel cross section area, a channel transmission of *T* = 0.199 is obtained. In other words, about 20% of photons impinging on the channel are transported in guided modes to the exit. Note that this experimental value accounts for the sum of all propagating modes, while the theoretical calculations in Figs. 2 ▸(*e*), 2 ▸(*f*) were plotted only for the fundamental mode *m* = 0, which has the highest transmission.

**Figure 6:**
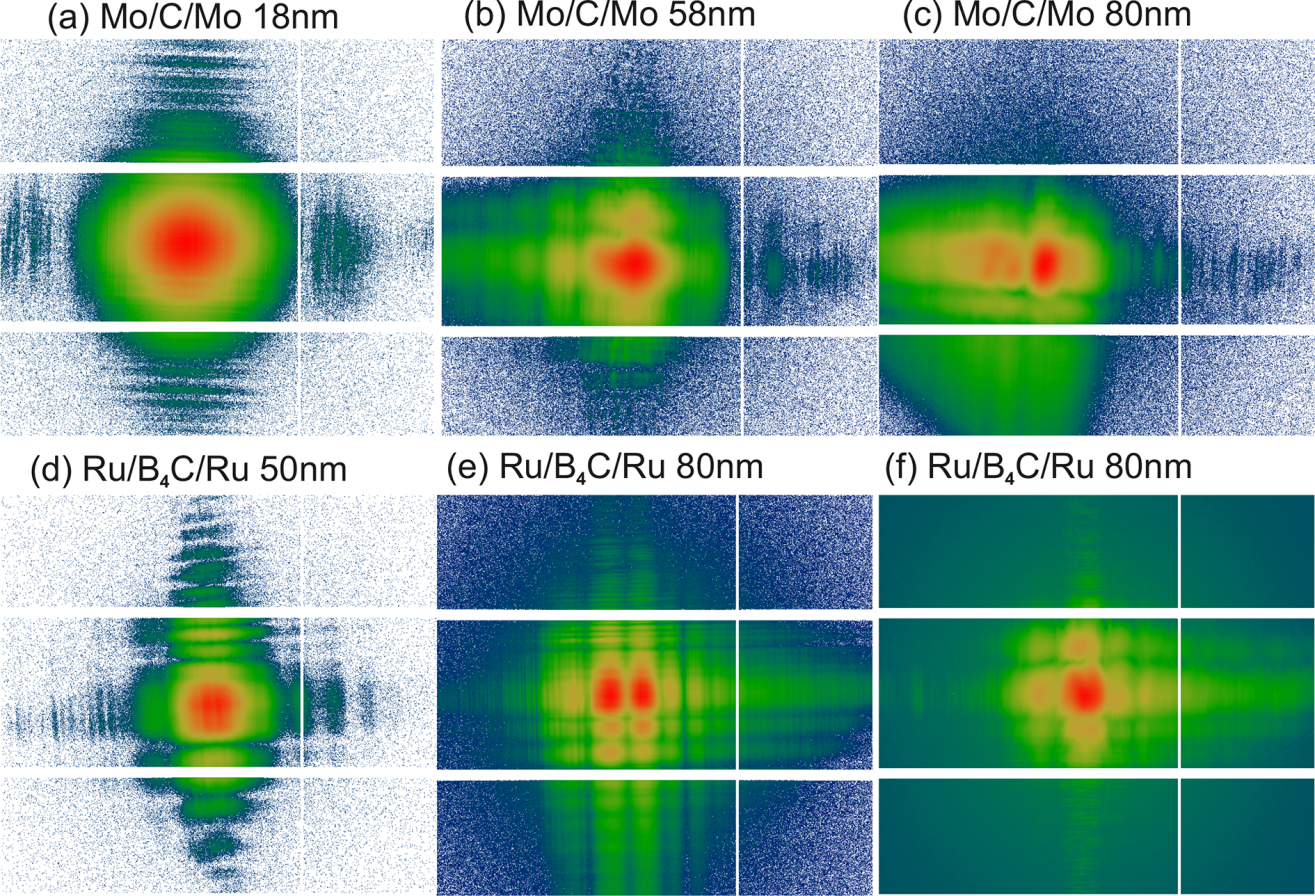
Far-field intensity patterns for different WGx, recorded by the Eiger detector at *E* = 13.8 keV. (*a*–*c*) Mo/C/Mo system with (*a*) *d* = 18 nm and *L* = 0.5 mm, (*b*) *d* = 58 nm and *L* = 0.5 mm, (*c*) *d* = 80 nm and *L* = 0.74 mm. (*d*–*f*) Ru/B_4_C/Ru system with (*d*) *d* = 50 nm and *L* = 2 mm, (*e*) *d* = 80 nm and *L* = 0.3 mm, (*f*) *d* = 80 nm and *L* = 0.74 mm. The WG intensities integrated over the detector area are (in photons s^−1^): (*a*) *I*_WG_ = 1.0 × 10^7^, (*b*) *I*_WG_ = 2.4 × 10^8^, (*c*) *I*_WG_ = 3.5 × 10^8^, (*d*) *I*_WG_ = 3.2 × 10^7^, (*e*) *I*_WG_ = 9 × 10^8^, (*f*) *I*_WG_ = 3.5 × 10^9^. Logarithmic color code: top (red) end of indicated color palette is mapped to (*a*) 5000, (*b*) 50000, (*c*) 80000, (*d*) 8000, (*e*) 3 × 10^5^ and (*f*) 1.2 × 10^7^ photons; the bottom end (blue) is mapped to one photon; 2 × 2 pixel binning for display. Scale bar: 200 pixels, corresponding to 15 mm.

Fig. 6 ▸ shows the far-field intensity distribution for different WGx, available at GINIX, including the new Ru/B_4_C/Ru system. Next to transmission and integrated flux, the visual inspection of the far-field pattern is important in view of imaging applications, since modal interference and stability matter for holographic illumination. It is advantageous, for example, to have a well developed maximum of the intensity around the optical axis in the forward direction. The background intensity due to radiative modes is also important. In particular for the WGb type, with Si as a cladding, the attenuation is insufficient when radiation is scattered out of the channel and traverses the silicon wafer only partially. In this case, the radiative modes are not damped over the total length *L* of the guide. The resulting coherent background radiation is emitted from the side of the WG channels and interferes in the far-field, forming circular fringes around the central modal interference. In contrast for the WGx type, semi-transparency for radiative modes is encountered, when the KB beam is coupled into one and not two 1DWG lamella and the thickness of a single 1DWG is insufficient to absorb the KB beam. Due to the smaller *d* values and accordingly lower number of modes, the WGx type shows a more pronounced pattern of modal interference, which changes with alignment, *i.e.* position of the WG entrance in the KB focus and photon energy *E*. The guiding layer thickness *d* determines the number of modes that can be supported by the WG, and which can interfere in the far-field. The patterns also depend on length *L*, photon energy *E* and coupling conditions, in particular the incidence angle of the front-coupled beam. The pattern of the *d* = 18 nm guide is the most homogeneous and has the highest NA. However, with *I*_0_ ≃ 10^7^ photons s^−1^ the WG intensity is too low for most applications. In contrast, the multi-modal far-field shown in (*e*) for a *d* = 80 nm guide has an integrated intensity of *I*_0_ ≃ 9 × 10^8^ photons s^−1^.

The extension of the WGx system at GINIX by the novel material combination Ru/B_4_C/Ru was motivated by the higher electron density of Ru, compared with Mo, and by the thin film quality achieved in sputter deposition. The performance can be assessed by comparing the intensity and transmission of the new WGx with that of the established Mo/C/Mo system, which has been available since the instrument began operation in 2010. While the intensities of WGx previously ranged between 10^8^ and 10^9^ photons s^−1^ (Krüger *et al.*, 2012 ▸; Töpperwien *et al.*, 2018 ▸), the exit flux for the best WGx has now increased by an order of magnitude, *e.g.* for Ru/B_4_C/Ru WGx with *d* = 80 nm (see Table 1 ▸). The flux increase benefits resolution and/or reduced acquisition time in holographic imaging, as further detailed in the supporting information.

**Figure 7:**
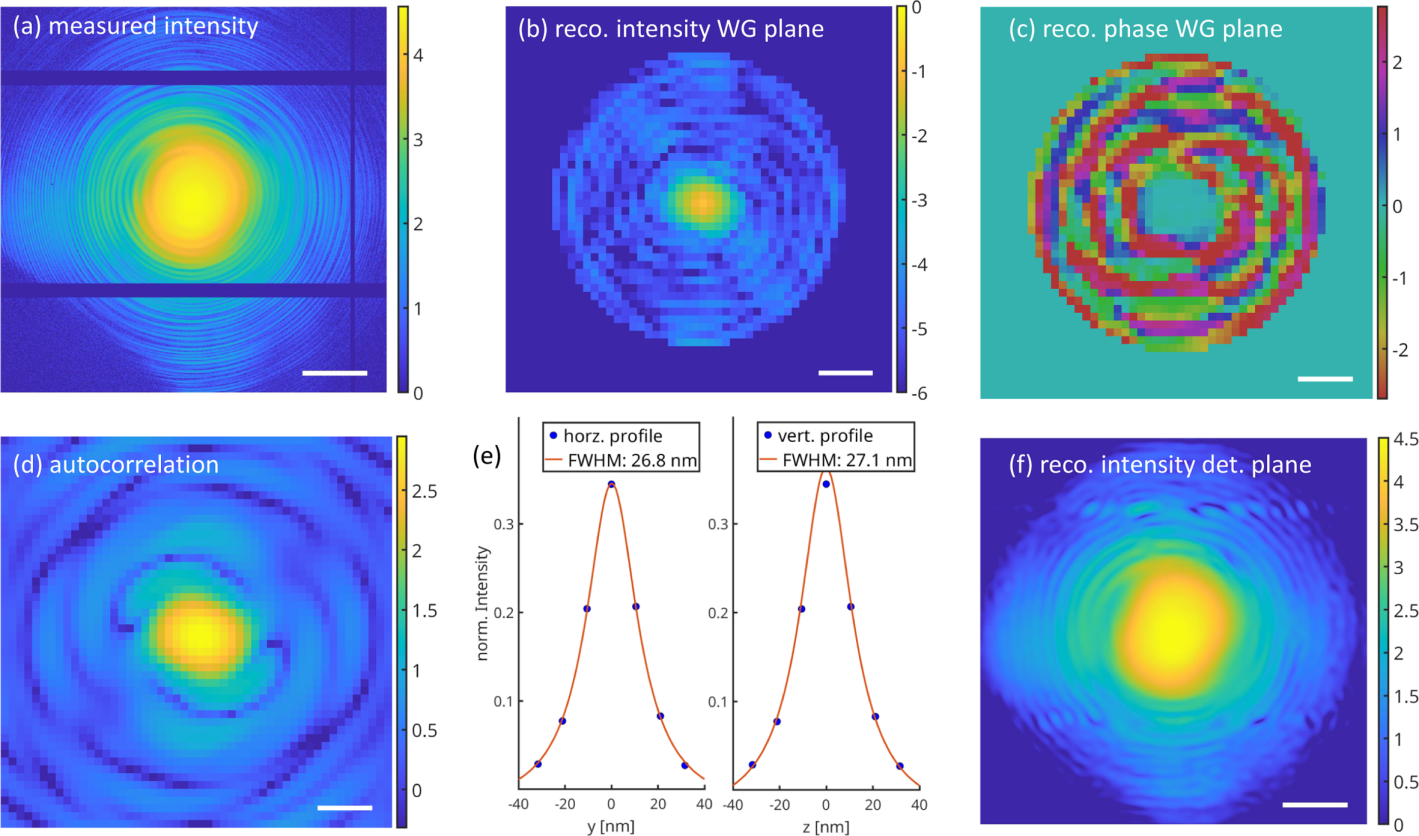
Reconstruction of WG wavefield by phase retrieval. (*a*) Far-field intensity distribution of *WGb-Ev3R3*, representing the measurements of phase retrieval, recorded by the Eiger detector at *E* = 8 keV, with *I*_WG_ = 9.82 × 10^8^ photons s^−1^. (*b*) Reconstructed intensity distribution in the WG exit plane |ψ(*x* = 0, *y*, *z*)|^2^ and (*c*) corresponding phase, after *N*_it_ = 500 iterations of the AP algorithm, with a circular support of *R* = 400 nm as a constraint (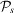). (*d*) Autocorrelation function of the near-field, obtained by inverse Fourier transform of the data in (*a*). (*f*) Reconstructed intensity in the detector plane (before projection onto the measurements). One can observe a reasonable filling of the inter-module gaps, which were masked out in the projection onto the measurements 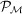. Color scale: logarithmic (base 10, photons s^−1^) in (*a*, *f*), linear in (*b*, *c*, *d*). Scale bar: 0.1 nm^−1^ in (*a*, *f*), 75 nm in (*b*, *c*, *d*).

In order to fully quantify the illumination, phase retrieval is performed, using alternate projections (AP), *i.e.* iterations of projection onto the measured intensities 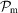 and projection onto the support 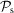 (support constraint) in the WG exit plane, which is a sufficient condition for WG phase retrieval under conditions of sampling specified by Krüger *et al.* (2010 ▸). In between the planes, wavefield propagation by Fraunhofer far-field diffraction implemented by the fast Fourier transform (FFT) and back-transform is used. The resulting near-field distribution in the WG exit plane ψ(*x* = 0, *y* = 0, *z*) fully defines the illumination function in phase and amplitude, since it can be propagated numerically to any position along the optical axis *x*, where the object is to be positioned. Fig. 7 ▸ exemplifies the phase retrieval step for characterization of the WG illumination. Note that the obtained spot size forming the secondary sources for holography is smaller than the channel width *d*. This is a result of multi-modal interference, and can also be observed in the WG simulations (see Fig. 1 ▸).

**Figure 8:**
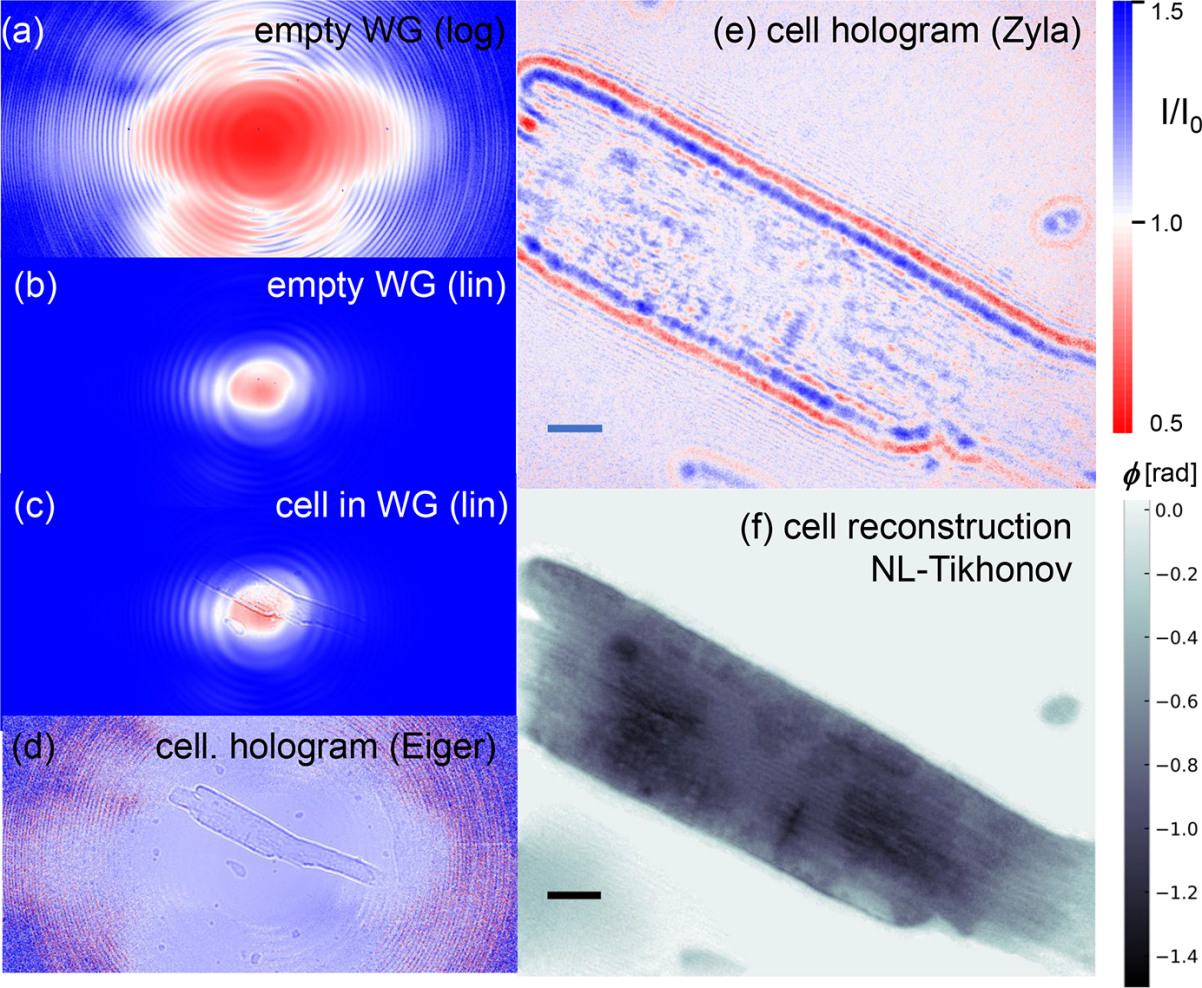
Alignment of a sample and holographic phase reconstruction, here illustrated for a cardiomyocyte cell, recorded with *WGb-Ev3R3*, channel #32, using (*a*–*d*) the Eiger4M pixel detector, and (*e*, *f*) the Zyla 5.5. HF (Andor). (*a*, *b*) The empty WG beam on a single module of the Eiger4M, in (*a*) logarithmic and (*b*) linear color scale (maximum set to 20000 photons). (*c*) The cardiomyocyte sample in the WG beam, again imaged by the same module of the Eiger 4M, linear color scale (maximum set to 20000 photons). (*d*) Same recording, after empty beam division, linear color scale between 0 and 3. (*e*) Same cell, nearly same position, but now imaged with the Zyla, again after empty beam division, linear color scale between 0.5 and 1.5. (*f*) Reconstructed object (phase), after phase retrieval by the non-linear Tikhonov scheme (Huhn *et al.*, 2022 ▸; Lohse *et al.*, 2020 ▸; Lucht *et al.*, 2024 ▸). Scale bars: 5 µm.

Finally, Fig. 8 ▸ illustrates alignment of an object in the WG beam and holographic imaging, here using the example of a cardiomyocyte (CM) cell. The CM was prepared from wildtype mice and mounted on a thin SiN-foil in a freeze-dried state, as done by Reichardt *et al.* (2020 ▸).

Since WG alignment and characterization are best performed by photon-counting pixel detectors to have a larger overview of the far-field pattern, as well as absolute photon counts, the first positioning and inspection of a sample is carried out with a photon-counting pixel detector, more specifically the Eiger4M detector at GINIX, before indirect detection with smaller pixel size is used for holographic imaging. The empty WG beam of *WGb-Ev3R3*, channel #32, is shown in (*a*) logarithmic and (*b*) linear scaling. The sample is then positioned in the FOV [see (*c*)]. Here a much larger FOV and a clearer view of the object is obtained after empty beam division [compare (*d*) with (*c*)]. The defocus distance *x*_01_, *i.e.* the distance between the WG exit and the center of rotation of the sample stage, on which the sample is positioned, is then adjusted to the desired FOV and zoom. The object position in the beam is controlled by moving motors and taking pictures from the command line, guided by the live display of the instrument’s control software. Magnification *M* = *x*_02_/*x*_01_ can be determined by automated macro commands which translate the object through the beam. Using a series of *x*_01_, both the distance between source (WG exit) and detector *x*_02_ and the defocus *x*_01_ can be checked against their nominal values, and recalibrated if needed. For the actual holographic recordings on which the object reconstruction is based, the pixel detector is replaced by a fiber-coupled sCMOS camera (Zyla 5.5 HF, Andor) with 2560 × 2160 pixels of 6.5 µm pixel size. The example shown in (*e*), (*f*) was recorded at *x*_01_ = 20 mm and *x*_02_ = 5.21 m, corresponding to *M* = 260.5 with effective pixel size px_eff_ = 25 nm. Together with the photon energy *E* = 8 keV, these parameters resulted in a Fresnel number of 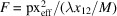 = 0.0002, in the deeply holographic regime. Note that for definition of *F* we take the pixel size as the characteristic object length scale, a convention that is useful in numerical image reconstruction, since it is the only parameter required for numerical wavefield propagation when using natural units. Accordingly, the acquisition recorded with the Eiger4M has a Fresnel number which is two orders of magnitude larger, *i.e.F*_*E*_ = 0.0266. Indeed, the image of the CM appears much less holographic, simply because the larger pixel size now averages over Fresnel oscillations. Image reconstruction is performed starting from the high-resolution hologram recorded by the Zyla camera. Estimating phase shift and attenuation, the single cell is well approximated by a pure phase object, but small ratios β/δ 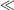 1 for the imaginary and real decrement of the index of refraction *n* = 1 − δ + *i*β can also be assumed for phase retrieval. First, a single-distance contrast transfer function (CTF) reconstruction is performed (Lucht *et al.*, 2025 ▸) using regularization parameters α_1_ = 0.0001 and α_2_ = 0.1, for the low and high spatial frequencies, with no constraints apart from homogeneity set to β/δ = 1/500. From the CTF reconstruction, it is then straightforward to generate a support mask. This mask is then added as a constraint set, and a non-linear Tikhonov solver (Huhn *et al.*, 2022 ▸; Lohse *et al.*, 2020 ▸; Lucht *et al.*, 2024 ▸) with 500 iterations is applied to compute the final reconstruction, shown in (*f*). The resulting phases are proportional to the projected electron density, ranging between −1.5 rad and 0 rad. Inner structures of the cells and notably to some extent the myofibrillar structure of the CM are resolved, as far as this is possible from a two-dimensional projection. Note, however, that the reconstruction shown in Fig. 8 ▸ is for an acquisition time of 1 s only, as is typical during an alignment scan.

In order to fully quantify the resolution obtained for the CM cell in a flux-dependent manner, we have recorded a series of 100 acquisitions, each of 1 s with corresponding empty images. We then performed Fourier shell correlation by splitting this acquisition in two halves, obtaining a half-period resolution of 98 nm, as detailed in the supporting information. In addition, we show how the resolution depends on integrated WG flux, underlining the relevance of a sufficient WG transmission.

## 5. WG beam splitters and interferometers

In the section above, WGs were presented as quasi-point sources for holographic imaging, which is their main purpose at GINIX. In the simplest design, they are straight channels. However, *WGb-Ev3R3* introduced in Fig. 5 ▸ already includes structures of a more complex design, notably tapered channels used to concentrate the beam by matching the WG entrance to the spot size of the focusing optics (Chen *et al.*, 2015 ▸). More general designs using an array of WG channels, and a concept of *X-ray optics on a chip* was demonstrated by Salditt *et al.* (2015*a* ▸). This included propagation in curved WG channels (Salditt *et al.*, 2015*a* ▸) and beam splitting for nano-interferometry (Hoffmann-Urlaub & Salditt, 2016 ▸). Multiplexed beamlets based on splitting and redirecting of beams in an X-ray WG chip could also serve as tailored illumination systems for more general holographic schemes, including for example different view angles and/or synthesis of high numerical aperture.

**Figure 9:**
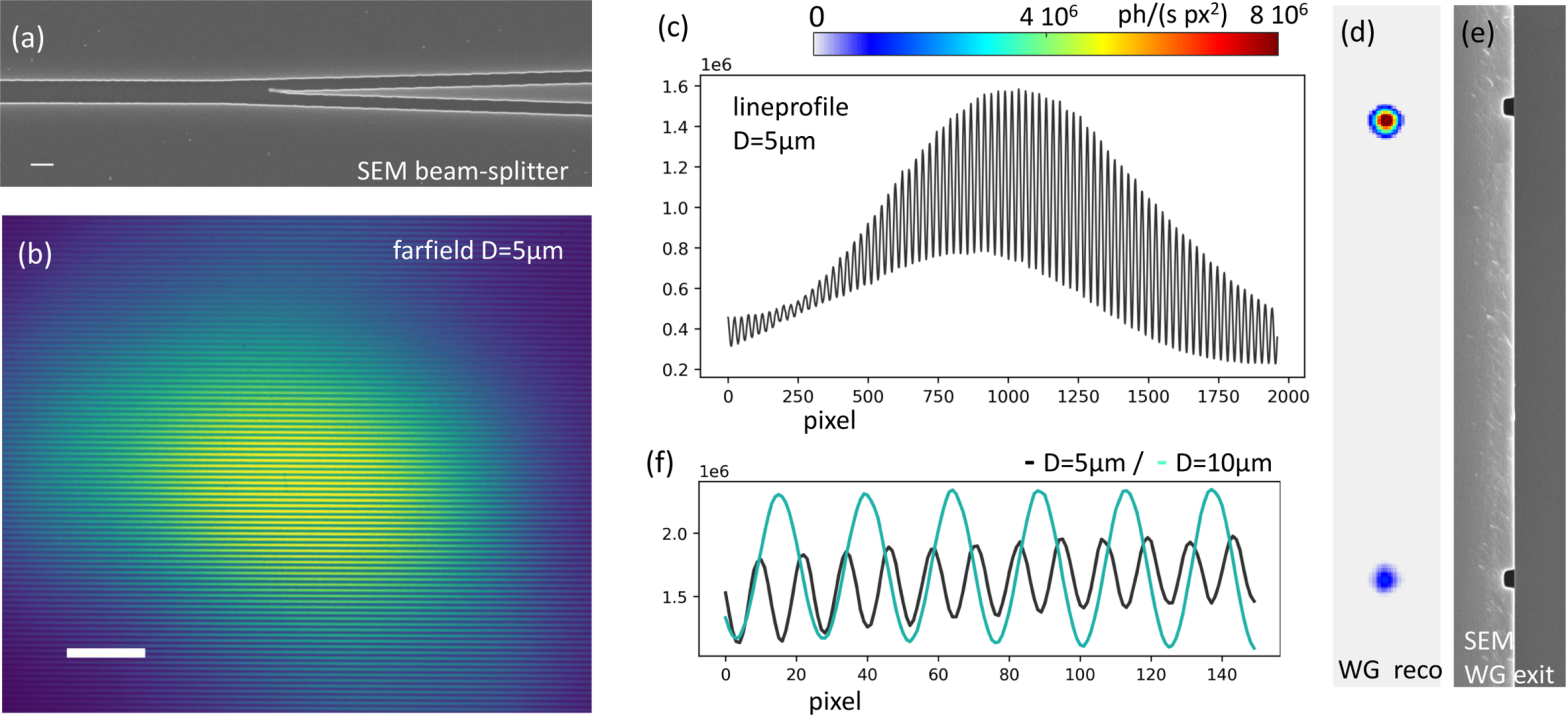
WG beam splitter and interferometer, which can be used to encode and measure the absolute phase of an object inserted in one of the two beams, and also serve off-axis holography or ptychography with highly diverse probes. (*a*) SEM image showing the region, where one channel splits into two (before wafer bonding). (*b*) Far-field pattern with double-slit-like interference for *D* = 5 µm. (*c*) Line-cut to assess the visibility of the interference pattern. Here, the two beams do not exhibit equal intensity, such that the destructive interference cannot reach zero. (*d*) Reconstruction of the near-field intensity in the WG exit plane, confirming the unequal intensity, and (*e*) corresponding SEM image of the WG exit showing the two channels. (*f*) The oscillation period of the interference pattern doubles for *D* = 10 µm.

Such advanced designs require lithographic fabrication techniques, *i.e.* the WGb type. WG beam splitters can be used for off-axis holography, where one of two coherent beams emanating from the WG exit face passes through the object, while the other one provides a reference for absolute measurements of phase. To this end, two diverging beams can be brought to interfere in the far-field. In this off-axis X-ray holography scheme the two beams emanate from the same X-ray WG, and are derived from a single WG channel by a splitter. In this way, high mutual coherence can be achieved also at a separation *D*, higher than the coherence length or focus of the incoming beam. Further, vibrations are avoided since the channels are placed on a single chip. A simple version of this off-axis X-ray holography scheme was realized (Fuhse *et al.*, 2006 ▸) without a true beam splitter. The incoming beam was coupled into two parallel channel WGs, and holographic reconstruction of a thin metal test wire was performed by back-propagation. Splitting of a single WG channel into two was first demonstrated by Hoffmann-Urlaub & Salditt (2016 ▸), but only for a rather small exit distance of *D* = 3 µm, and without any object in the beam. Furthermore, the visibility of the double-slit interference patterns and quality of the far-field pattern were limited due to fabrication challenges. In Fig. 9 ▸, we present WG channels for *D* = 5 µm and *D* = 10 µm based on the improved fabrication schemes presented in the previous sections, and show a far-field interference pattern with higher visibility, flux and overall quality.

**Figure 10:**
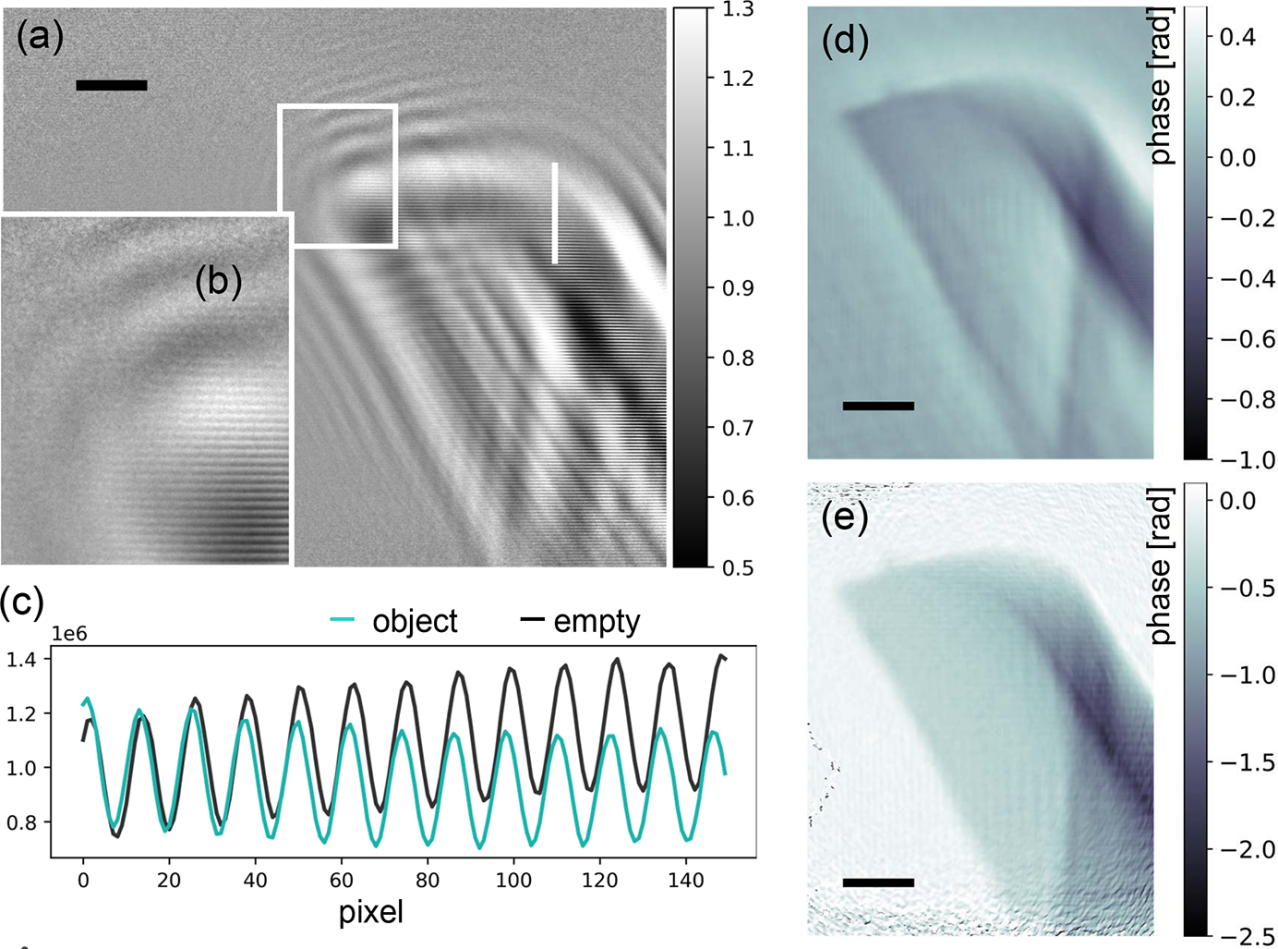
Off-axis holography with double WG interferometer. (*a*, *b*) Empty beam divided hologram. The double-slit-type fringes present in both acquisitions, with and without object, disappear when no object is present. In contrast, within the hull of the object hologram, fringes remain after the division of object and empty beam, since the phase shift introduced by the object interferes with the reference beam, shifting the fringes proportionally to the local object phase. (*c*) A line-cut along the white line in (*a*) through empty beam (black) and object image (cyan) directly shows the attenuation as well as the phase shift. When no object is present, the two signals are identical. In detector pixels receiving object signal the intensity is attenuated and the oscillations are shifted in phase. (*d*) CTF reconstruction of the hologram in (*a*), after blurring of projection and empty beam in order to remove the fringes. (*e*) Off-axis reconstruction based on back-propagation (Fuhse *et al.*, 2006 ▸), showing the expected range of negative phases and stabilized low spatial frequencies. Scale bars: 1 µm.

Fig. 10 ▸ shows off-axis holography recorded with *WGb-Ev3R3*, structure #58, a beam splitter with two exit arms separated by *D* = 10 µm. A thin glass capillary with a broken tip is positioned into one of the WG beams at *x*_01_ = 3.39 mm, before the beams broaden by diffraction, overlap and interfere. With a detector distance *x*_02_ = 5.03 m, the resulting magnification is *M* = 1527, and with photon energy *E* = 8 keV we get *F* = 2.55 × 10^−5^. The local phase shift between object beam and offset beam is proportional to the projected electron density, as in standard two-arm interferometry and and can be quantified from the shifts of the fringes. Since the two WG beams are emanating from quasi-point sources, the object’s holographic image is magnified, and the local phase shifts are encoded in the horizontal (double-slit) fringes. The locally varying phase reflects the optical path length through the object in different locations of the image. The object-induced phase shift can be shown by empty beam division and the remaining fringes where the object is present [Figs. 10 ▸(*a*), 10 ▸(*b*)], as well as from line-cuts [Fig. 10 ▸(*c*)]. Since the illumination is inconsistent with standard phase retrieval, for example by CTF, we first convolve the hologram by a Gaussian kernel (σ = 5 px) before applying the CTF reconstruction. The resulting image is shown in Fig. 10 ▸(*d*), for regularization parameters α_1_ = 0.01 and α_2_ = 0.1, for the low and high spatial frequencies, respectively, and using the ratio β/δ = 1/76 from literature values of X-ray optical indices for SiO_2_. No further constraint is used. Due to the optical nature of the tip as a rather thick object, as well as the large gradients of the phase, linear phase retrieval approaches such as CTF must fail, and the range of the resulting phase is incorrect. In contrast, a simple-minded off-axis reconstruction following Fuhse *et al.* (2006 ▸) results in much better stabilized phase retrieval, including low spatial frequencies which are often problematic in standard propagation-based phase-contrast imaging without a reference beam. Note that the phase values here are quite uniform in areas where the beam traverses only a single glass surface. This typically cannot be achieved for inline-holographic reconstructions of single-distance acquisition without a support constraint.

For the off-axis reconstruction by back-propagation, we used a numerical illumination corresponding to an oblique plane wave with a phase periodicity given by the double-slit oscillations. Importantly, this type of very simple reconstruction has no further free parameters, apart from the experimentally known Fresnel number *F* and oscillation period, directly measured from the empty beam on the detector. In future, more powerful iterative phase retrieval schemes should be worked out, initialized by the single one-step reconstruction based on back-propagation.

## 6. Summary, conclusions and outlook

In summary, we have presented recent progress in WG optics at the GINIX instrument, including the commissioning and characterization of new WGs, holographic probe preparation and retrieval, and finally WG interferometers for off-axis holography. Considerable efforts are made to control the properties of the illumination probe since it directly affects the quality of holographic imaging and its dose efficiency. Most important is a small focal size which warrants a large NA of the holographic illumination and hence high resolution. Apart from NA and resolution, sufficient coherence and smooth wavefronts are essential (Bartels *et al.*, 2015 ▸; Krenkel *et al.*, 2017 ▸; Hagemann *et al.*, 2017 ▸; Hagemann & Salditt, 2018 ▸). Since X-ray nano-focusing is associated with significant wavefront distortions, the idealizing assumptions on the probe which are necessary in pre-processing (in particular, empty beam division) and phase retrieval (full coherence) are often not satisfied. When using empty beam division in pre-processing, for example, wavefront distortions lead to reduced resolution and image quality (Homann *et al.*, 2015 ▸; Hagemann *et al.*, 2014 ▸). To mitigate these effects, in particular the strong KB wavefront distortions visible as stripe artifacts, WG optics have been implemented at GINIX for coherence, spatial and wavefront filtering of the probe (Salditt *et al.*, 2008 ▸; Osterhoff & Salditt, 2011 ▸; Krüger *et al.*, 2012 ▸; Chen *et al.*, 2015 ▸; Salditt *et al.*, 2015*a* ▸). Since the initial installation, the WG optics have been continuously improved. The reconstructed near-field distribution of WGb-type optics still shows residual transmission of radiative modes, visible as background in the WG exit plane. In future, this could probably be avoided by replacing Si with Ge or GaAs. For the WGx-type optics, radiative modes are less of a concern. However, WGb-type optics are required for WG interferometers and for designing more complex illumination fields for off-axis holography. Here we showed that even a very rudimentary implementation of off-axis holography significantly stabilized phase retrieval, since the second beam which does not traverse the object provides an absolute phase reference. Independent of WG design and fabrication, remaining challenges at GINIX are related to the instabilities of the mechanical setup and thermal drift. When the holographic probe is non-stationary, both empty beam division and probe-informed reconstruction are compromised. Vibration control and thermal stabilization are hence of highest priority for an instrumental upgrade.

It is also of interest to place inline nano-holography and holo-tomography with WG illumination at GINIX into a broader context of other holo-tomography instruments. For this purpose, beamline ID16a at the ESRF can be considered as a benchmark instrument. While GINIX uses a compound system consisting of a single surface fixed-curvature KB for focusing and a WG optic for spatial and coherence filtering, ID16a achieves sub-30 nm spot size in ‘one go’ based on flexible curvature multilayer KB mirrors. It offers two orders of magnitude higher flux, and currently the highest resolution of all full-field nano-holography instruments, in particular after the EBS upgrade of the ESRF. However, for recordings with highest coherence, the spatially fully coherent beam of the GINIX instrument, and the longitudinal (temporal) coherence resulting from the Si(111) monochromator at P10 offer certain advantages. Furthermore, the KB mirror surfaces at ID16a introduce strong wavefront artifacts, which can hamper empty beam division and limit image quality (Robisch *et al.*, 2016 ▸). Finally, the impressive stability of the ID16a beam is also related to its in-vacuum sample environment, which is incompatible with imaging of biological samples in solution. In contrast, the sample environment of GINIX is in air. The instrument offers unique opportunities for nanoscale imaging of unstained and even hydrated biological tissue at moderately low dose. For better stability and higher flux, however, an instrumental redesign along with the planned PETRA IV upgrade is imperative.

Finally, we want to briefly address what type of WG optic would be suitable for holographic imaging at the future coherence application beamline (CAB). The proposed PETRA IV upgrade will result in a 100–1000-fold higher brightness as compared with today. Due to the unprecedented resolution potential arising from this emittance reduction and brilliance gain, there is a need to reconsider the focusing optics, in particular for high-resolution 3D imaging by holo-tomography. The recent progress in high-NA focusing optics, for example based on multilayer Laue lenses (MML), has created new opportunities to tailor the illumination function, and unlike today the WG optics may not be required to reduce the focal spot size, *i.e.* the virtual quasi-point source for holographic imaging. In particular, high-NA MLL optics (Bajt *et al.*, 2018 ▸; Zhang *et al.*, 2024 ▸) in combination with pixel detectors seems to be a promising optic for holographic imaging. However, the option to filter the wavefront and to decouple the illumination from upstream beamline optics by modular WG optics could still be useful. Even if the WG is no longer needed to reach a small focal spot, it helps to block background radiation, to eliminate tails of a focusing optic as well as higher diffraction orders which are unavoidable in diffractive optics. To this end, the modular and flexible design of the GINIX instrument can be used for pathfinding experiments with different optical setups, helping to specify the optical concept and layout of the CAB at PETRA IV. In particular, the WG exit plane provides a plane in the optical system where the probe can be made extremely compact, providing a strong constraint for probe-informed phase retrieval. Ideally, for this purpose, the WG portfolio should be extended. The currently used WGb type in Si should be replaced by materials of higher critical angle θ_c_. Notably, Ge and GaAs are suitable materials, since wafers of sufficient surface quality are available and wafer bonding is possible. However, when the NA of the prefocusing optics becomes higher than that of the WG, the WG would limit the NA. This happens when the Bragg angle θ_*n*=1_ corresponding to the first order of the diffractive optics exceeds θ_c_ of the WG material used, or equivalently when the outermost zone width δ*r*_*N*_ ≥ *W*, where *W* is the critical WG width introduced above.

The SRH approach (Soltau *et al.*, 2021 ▸), on the other hand, does not rely on the NA of the central cone of the holographic probe. Instead, holographic and diffractive signals are reconstructed jointly and the NA is limited by the highest signal that can be recorded in the tails of the probe and beyond. Presently, however, the residual transmission of radiative modes and the background seem to dominate such diffractive signals. For SRH, longer WG channels with more absorption of radiative modes may therefore be a good choice. The WG diameter *d* does not have to be extremely small, since the NA of the WG no longer limits the resolution. Note that *d* ≫ *W* can ensure high modal transmission, and hence high flux which is required to obtain sufficient diffractive signals. In contrast to conventional holo-tomography, where the hologram is first divided by the empty beam, SRH can also exploit signal in the tails of the far-field distribution. It therefore becomes of interest to design the WG optic not only in view of high *I*_0_, large NA but also in terms of the intensity distribution in the far-field tails (see the supporting information).

Aside from holography and full-field coherent imaging, WG optics can also serve as a diagnostic tool for a nano-focus optic. While ptychographic reconstruction of the focal or near-focal field distribution can be used to characterize a fully coherent probe (Kewish *et al.*, 2010*a* ▸; Kewish *et al.*, 2010*b* ▸; Schropp *et al.*, 2010 ▸; Guizar-Sicairos *et al.*, 2010 ▸; Guizar-Sicairos *et al.*, 2011 ▸; Mastropietro *et al.*, 2011 ▸; Hönig *et al.*, 2011 ▸; Giewekemeyer *et al.*, 2013 ▸; Giewekemeyer *et al.*, 2014 ▸; Wilke *et al.*, 2012 ▸; Wilke *et al.*, 2013 ▸; Wilke *et al.*, 2014 ▸), partially coherent beams such as most of the current KB beams are more challenging to characterize. Multi-mode ptychographic reconstruction can come to the rescue for probe reconstruction but these approaches quickly reach their limit when the number of modes becomes too high (Thibault & Menzel, 2013 ▸; Giewekemeyer *et al.*, 2013 ▸; Hagemann & Salditt, 2017 ▸). In this case, WG optics are useful as a nanoscopic slit system to map out the intensity distribution in and around the focal plane. In future, this may even be extended to a full characterization by exploiting approaches of coded aperture, using step scans with WGs translated and rotated through a focus, and a forward operator taking into account the full coupling conditions. Finally, WG beam splitters with multiple beams can also help in the design of diverse probes for ptychography. Note that when an object is placed in the region where the two beams interfere in the near-field, it is illuminated by a highly structured probe, with strong gradients.

More generally, WGs offer unique optical functionalities for nano-optics, including synchrotron and free-electron laser (FEL) radiation. Propagation in empty WG channels is nearly free of dispersion down to ultra-short pulse widths in the range of 0.1 fs (Melchior & Salditt, 2017 ▸). Miniaturized beam splitters with attosecond delay could be realized by splitting of a pulse and one beam propagating in a curved channel with longer path length. Splitting of one incoming beam into two can also be achieved by X-ray WGs in resonant beam coupling geometry, based on a giant Goos–Hänchen effect (Zhong *et al.*, 2017*a* ▸). In this case, one beam is displaced along the surface with respect to the other. Most interestingly, WG nano-optics can be used to control the interaction of the modal field with resonantly excited emitters (Lohse *et al.*, 2025 ▸).

## 7. Related literature

The following references, not cited in the main body of the paper, have been cited in the supporting information: Chen (2006 ▸); Fuhse (2006 ▸); Gloge (1971 ▸); Goodman (2020 ▸); Hoffmann-Urlaub *et al.* (2016 ▸); Lee *et al.* (2023 ▸); Mai *et al.* (2013 ▸); Osterhoff (2012 ▸); Osterhoff & Salditt (2009 ▸); Salditt *et al.* (2011 ▸); Salditt & Osterhoff (2020 ▸).

## Supplementary Material

Supporting information with additional graphics and material. DOI: 10.1107/S1600577525011567/tol5017sup1.pdf

## Figures and Tables

**Table 1:** WGx-type WGs available at GINIX: materials, labels, guiding layer thickness *d*, optical length *L*, exit flux *I*_0_ and transmission *T* The numbering refers to the WGx list at the instrument (run111). All values have been measured at 13 keV, with a measured KB flux of 1.26 × 10^11^ photons s^−1^. The geometric mismatch of KB focal spot size and *d* has been taken into account. The low transmission of the 2 mm-long WGx reflects the fact that this WG was designed for an experiment at 40 keV. However, despite the longer *L*, WGx No. 1 shows a much higher *T* than No. 7, which may be explained by the fact that the coupling between the two WG lamellae is not perfect.

WGx type	WGx No.	*d* (nm)	*L* (mm)	*I*_WG_ (photons s^−1^)	*T* (%)	Remark
Mo/C/Mo	2	18	0.5	9.2 × 10^6^	0.037	Old
Mo/C/Mo	5	58	0.6	2.93 × 10^8^	0.05	Old
Mo/C/Mo	6	80	0.74	4.44 × 10^8^	0.04	Old
Ru/B_4_C/Ru	4	50	2	2.1 × 10^7^	0.011	New
Ru/B_4_C/Ru	7	80	0.3	1.03 × 10^9^	0.09	New
Ru/B_4_C/Ru	1	80	0.74	4.86 × 10^9^	0.44	New

## Data Availability

All data on the WG optics presented here are available upon reasonable request, and by default also to GINIX users along with the WGs themselves.
