# Supplementary material for: X-ray waveguide optics at GINIX/P10 PETRA III: recent progress and future directions
Source: J Synchrotron Radiat. 2026 Feb 10;33(Pt 2):298–313. doi: 10.1107/S1600577525011567 (PMC12948026; doi:10.1107/S1600577525011567)
Supplement: Supplementary file 1 [file s-33-00298-sup1.pdf]

JOURNAL OF SYNCHROTRON RADIATION

Volume 33 (2026)

Supporting information for article:

X-ray waveguide optics at GINIX/P10 PETRA III: recent progress and future directions

Tim Salditt, Paul Meyer, Leon Merten Lohse, Jens Lucht, Jakob Soltau, Neele Kozák, Mike Kanbach, Markus Osterhoff and Fabian Westermeier

X-ray waveguide optics at GINIX/P10 PETRA III Supplementary Material

Tim Salditt [a], Paul Meyer [a], Leon Merten Lohse [a], Jens Lucht [a], Jakob Soltau [a], Neele Kozák[a], Mike Kanbach[a], Markus Osterhoff [a], and Fabian Westermeier [b]

[a]Institut für Röntgenphysik, Georg-August-Universität Göttingen, 37077 Göttingen (Germany)
[b]Deutsches Elektronensynchrotron (DESY), Notkestrasse 85, 22607 Hamburg (Germany)

1 Progress in WGb fabrication

The quality of the WGb channels depends on all of the fabrication steps, including pattern generation by electron beam lithography (EBL), pattern transfer into the wafer by reactive ion etching (RIE), and finally the wafer bonding process at high temperatures. Straightness of channel interfaces and microscopic interfacial roughness are both of concern, and the effects of both have been studied before by numerical simulation, for example in (Osterhoff, 2012). Compared to the previous protocol and fabrication (Hoffmann-Urlaub *et al.*, 2016; Hoffmann-Urlaub, 2016), which was all carried out at the institute for X-ray physics (IRP), the EBL and RIE steps for Si WGb are now ordered commercially (Eulitha GmbH, Switzerland), and only wafer bonding and dicing is carried out inhouse. This has resulted in improved interfacial quality, see the side-by-side comparison in Fig.1, showing SEM micrographs of WGb, fabricated by the previous (Hoffmann-Urlaub & Salditt, 2016) and the new protocol.

2 Germanium waveguides: beam induced damage

When waveguides are positioned in the intense focus of a KB optic, problems of radiation induced reactions can arise, in particular when the waveguides are kept in air such as at the `GINIX` endstation. Ozone production and free-radical induced photo-reaction in particular of oxides are of concern. For silicon WGs, even after many days of beamtime with typical photon flux densities around $10^{11}/(s\ (250nm)^2)$, the transmission and far-field pattern seem little affected by extended irradiation, eventhough some deposition or material, presumingly organics, can be observed by SEM after prolonged use. Contrarily, Ge WGs were found to deteriorate in the beam, over a few days or even some hours. While we have not investigated this systematically, based on the observed X-ray transmission and mode pattern, this transformation seems to happen suddenly at some point after prolonged irradiation, rather than gradually. When using photon energies above the germanium

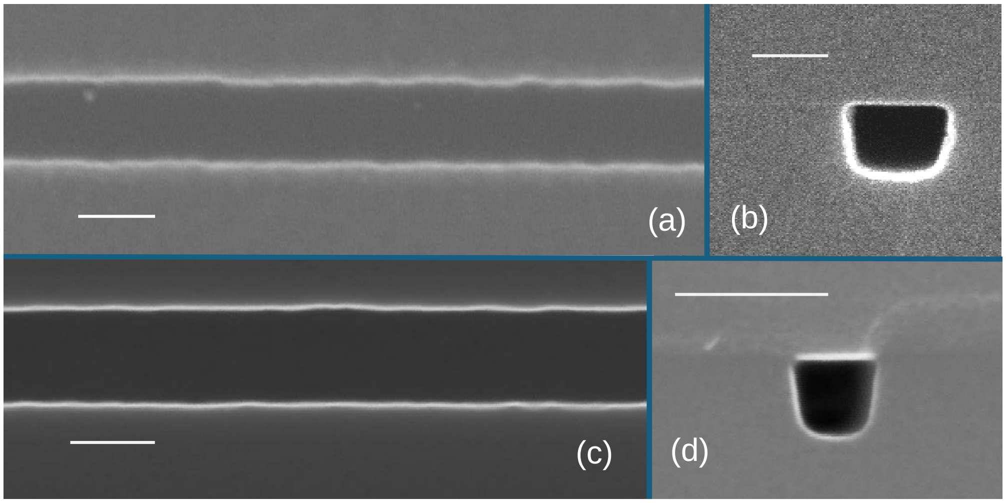

Figure 1: Progress in fabrication of Si WGb. (a,b) SEM micrographs of a WGb channel (a) before (top view), and (b) after bonding (front view), of the IRP inhouse protocol. (c,d) Same for a new Si WGb. Better side wall definition and interface quality can be achieved, even for smaller channels, such as in (d). Scale bars: 100 nm .

K-edge, the effect is accentuated. It seems likely that X-ray induced reactions of oxides play a major role in this process, and it remains to be checked whether this also occurs when the waveguide is kept in ultrahigh vacuum. A first case of particularly badly damaged wafer with Ge channels is presented in Fig.2. Note that the use of Ge wafers for attenuation of the beam in the WGx type did not suffer from such effects, while the effects were prominent in lithographically-defined and RIE etched channels of the WGb type, making it likely that surface composition and chemistry play a major role, including prior treatment of wafer surfaces.

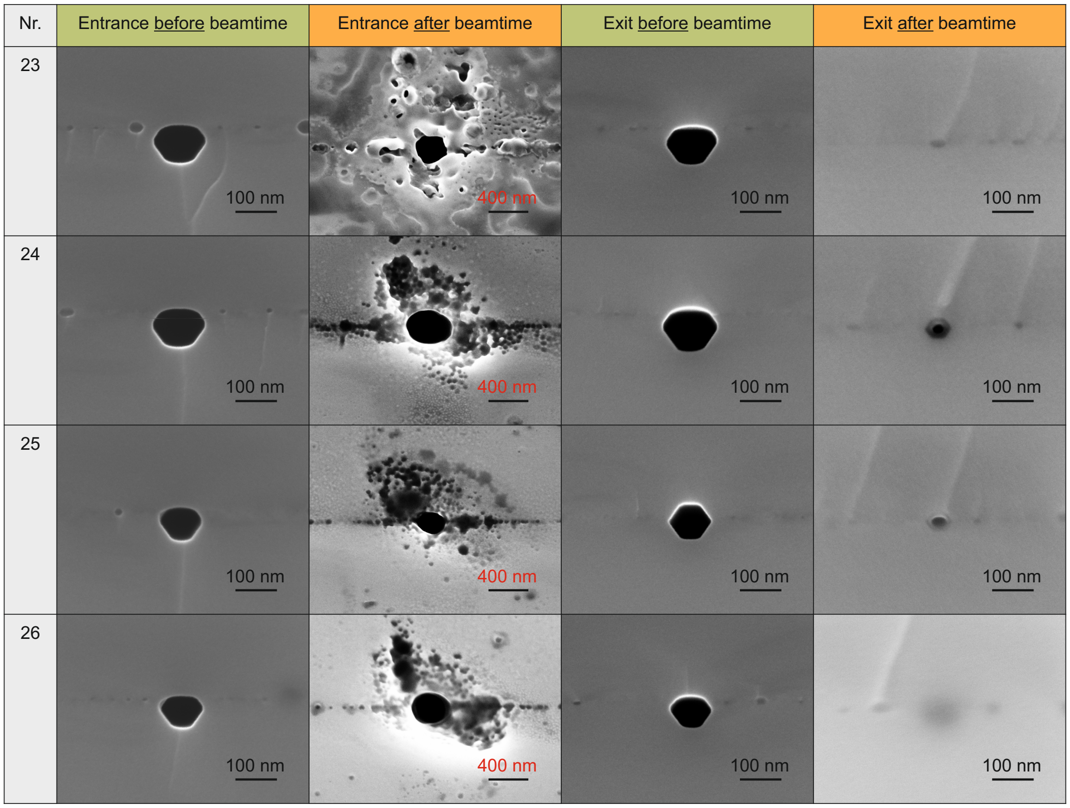

Figure 2: Beam-induced reactions in Ge waveguides. SEM images of *Ge* channels which were kept in the KB focus for an extended period (several hours up to several days). SEM images are shown for the entrance and exit face of four numbered channels which were all positioned on the same chip, before and after the beamtime. While surface roughness and morphology has severely degraded around the channel entrance, the exit channel seems to have narrowed, possibly by growth of oxides.

3 Resolution as function of accumulated WG flux

In the main MS, we showed the hologram and reconstruction of a cardiomyocyte obtained after a 1 s (one frame) acquisition with the fibre-coupled camera. For nanoscale resolution such single image aquisitions are often not sufficient, and subsequent images with accumulated signal are required. The relevance of high WG exit flux and a stable far-field derive from the fact that due to thermal drift and of the optics and the sample, the resolution does not always increase with the number of acquisition N_{acc} as one would expect for pure Poissonian noise (and in the absence of dark current and drift). To this end, it is important to correlate and shift the individual frames, before summing them up. In Fig.3, we show that while sub-100nm resolution is obtained from Fourier shell correlation (FSC) for $N_{acc} = 50$, the increase in resolution in this case is marginal for $N_{acc} \geq 15$. While the reduced noise results in higher resolution when initially increasing the

number of accumulations from $N_{acc} \geq 1$, saturation behaviour as well as non-linear behaviour of the phase retrieval (see red curve) can play a role. The resolution dependence on N_{acc} should in fact be studied in more detail in future in order to better understand under which circumstances signal accumulation is possible in practice.

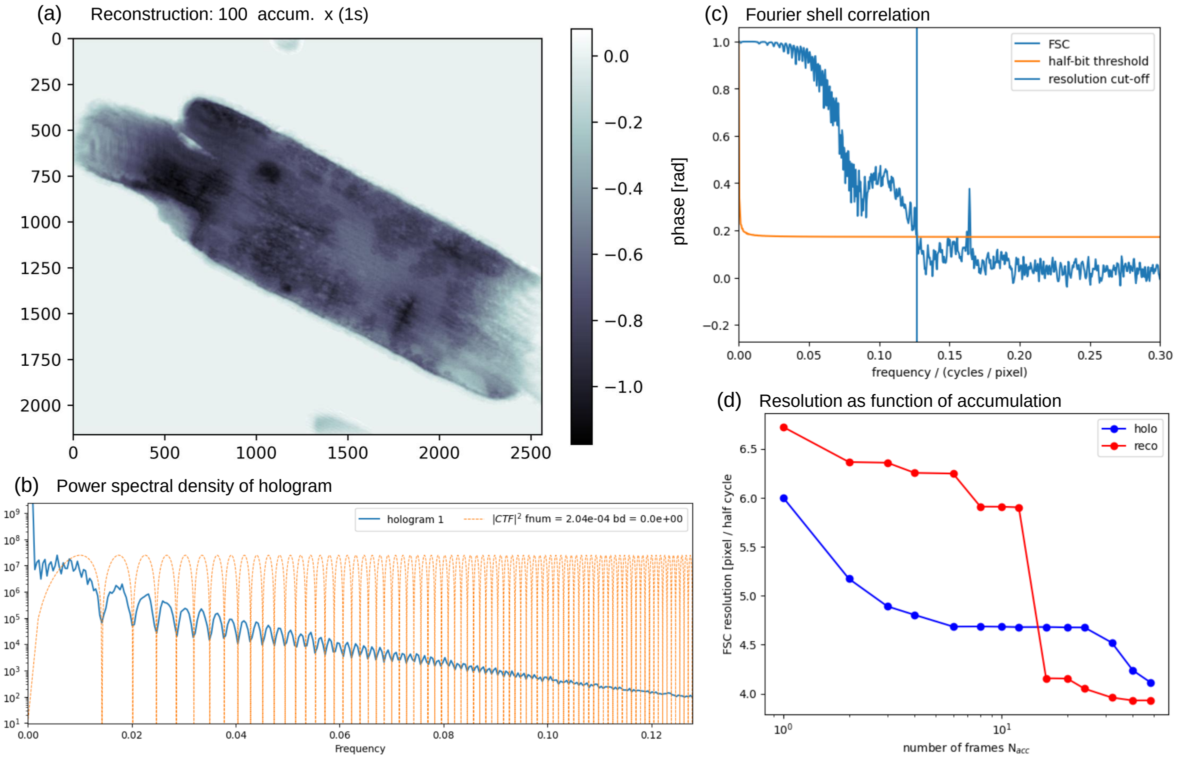

Figure 3: Quantification of image resolution for holographic imaging of the cardiomyocyte (see main manuscript). (a) Reconstruction after phase retrieval by the non-linear Tikhonov algorithm with an additional support constraint. The total exposure time was 100s, partitioned into 100 frames (100 × 1s). (b) Power spectral density of the hologram shown in (a). The high coherence of the waveguide illumination and the small Fresnel number F result in 50 visible oscillations of the phase contrast transfer function (CTF). (c) Fourier shell correlation (FSC) curve obtained from correlation of two reconstructions computed after splitting the dataset into two (two series of 50 × 1s exposure). Note that holograms *and* empty images were different for the two reconstructions. The crossover with the 1-bit resolution curve indicates a half width (HWHM) resolution of 98nm. (d) FSC resolution of the hologram (blue) and the reconstruction (red), as a function of the number of frames (in each one of the correlated datasets).

4 Waveguide Far-field Distribution

In the outlook section of the main manuscript, we briefly addressed WG optics for super-resolution holography (SRH) (Soltau *et al.*, 2021). In this approach, the hologram is not divided by the empty

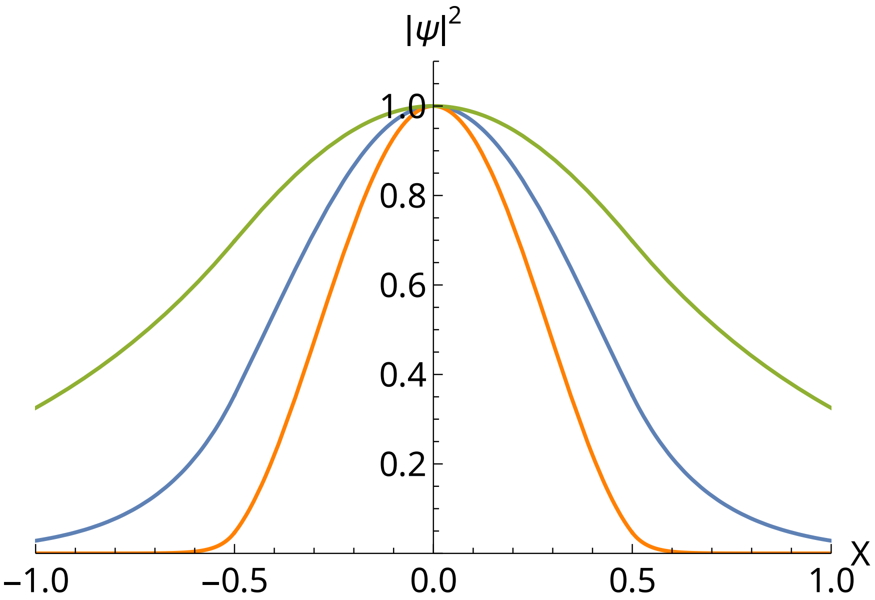

Figure 4: PWG/near-field. Normalized intensity of the fundamental mode in the PWG analytical model as a function of radial distance $X = x/d$, for different unitless potentials $V = 1\pi$ (blue), $V = 2\pi$ (orange), and $V = 0.44\pi$ (green). The blue curve corresponds to the case where the fibre becomes mono-modal, i.e. at the cutoff point $V_{sm} = \pi$ for the next higher mode. For $V < V_{sm}$ only the fundamental mode can propagate as a guided mode. The fraction of intensity located in the core, the so-called confinement factor is 0.86 at the cutoff point, see the blue curve. Higher V (orange) confine better, lower V (green) result in pronounced tails residing in the cladding.

beam as in conventional inline holography, where the signal is exploited only within the central NA of the holographic probe. Instead, signal can also be exploited from the tails of the probe and – if present – also from the diffractive signals out of the central lobe of the WG beam, forming a joint input for the reconstruction. Correspondingly, the overall resolution is no longer limited the width of the central cone, i.e. the region where the empty beam division is performed, corresponding roughly to the NA of the WG beam. It rather depends on the highest lateral momentum transfer q, where signal is still recorded on the (pixel) detector. In this case, the decay of the WG far-field intensity, i.e. the tails of the beam also becomes important design properties. For example, it may then be more important to reach high intensity and very low background of radiative modes than high NA. To gain an understanding of the far-field lineshape, the parameter space, and the underlying scaling behavior, analytical treatment can be used, applied to the case of hard x-rays (Salditt & Osterhoff, 2020; Fuhse, 2006; Osterhoff & Salditt, 2009). In (Osterhoff & Salditt, 2009) the far-field distribution was computed for the the fundamental mode of a planar waveguide at smallest width $d = 0.44W$, corresponding to to the highest NA. Here we extend this to a more general choice of d, and W, both for planar and for cylindrical WGs, using the theory of weakly guiding fibres (WGF).

4.1 Planar waveguides (PWG)

We consider a symmetric slab waveguide with refractive index given by a one-dimensional index profile $n(x)$ independent of y, and guiding direction z. The stationary wave equation (Helmholtz equation) can then be written as

$$\left(\frac{\partial^2}{\partial x^2} + \frac{\partial^2}{\partial z^2}\right)\psi(x,z) + k^2 n^2(x)\psi(x,z) = 0 \; . \tag{1}$$

Guided waves take the form

$$\psi_m(x,z) = u_m(x)\exp(i\beta_m z) \; , \tag{2}$$

where the modes $u_m(x)$ are found as analytical solutions of the reduced wave equation (Eq.(2) of the main manuscript). If the imaginary part of the refractive index can be neglected for mode formation ($Im(n) \simeq 0$), the propagation constant β_m of mode m is real-valued. Assuming further a step index profile with a guiding core of index n_1 and a cladding $n_2 < n_1$

$$n(x) = \begin{cases} n_1 & \text{if} - d < x < 0 \\ n_2 & \text{else} \; , \end{cases} \tag{3}$$

the ansatz for the field distribution of the mode can be written as

$$\psi(z) = \begin{cases} Be^{-\gamma_m z} & \text{for } z \geq 0 \\ B\cos(\kappa_m z) + C\sin(\kappa_m z) & \text{for } 0 > z \geq -d \\ (B\cos(\kappa_m d) - C\sin(\kappa_m d))e^{\gamma_m(z+d)} & \text{for } z < -d \; , \end{cases} \tag{4}$$

with mode parameters $\kappa_m = \sqrt{n_1^2 k^2 - \beta_m^2}$ and $\gamma_m = \sqrt{\beta_m^2 - n_2^2 k^2}$ (in units of inverse length). Requiring continuity of the field and the derivative at the interfaces, the allowed values κ_m are given by the zeros of the transcendental equation

$$\tan(\kappa d) = \frac{2\kappa\gamma}{\kappa^2 - \gamma^2} = \frac{2\kappa d\sqrt{(V^2 - (\kappa d)^2}}{(\kappa d)^2 - [V^2 - (\kappa d)^2]} =: F(\kappa d), \tag{5}$$

where the waveguide parameter V quantifies the strength of the guiding potential (i.e the 'depth' of the potential well)

$$V := \sqrt{n_1^2 - n_2^2}\, kd \approx \sqrt{2\delta_2 - 2\delta_1}\, kd \; , \tag{6}$$

and the decay parameter in the cladding is then determined from κ_m by

$$(\gamma_m d)^2 = V^2 - (\kappa_m d)^2 \; . \tag{7}$$

The far-field distribution can then be computed by analytical Fourier transform as a function of normalized momentum transfer $Q = qd$.

$$\tilde{\psi}(Q) = 2d \left(\frac{A\ e^{-\Gamma/2}}{\Gamma^2 + Q^2} \ (\Gamma \cos[Q/2] - Q \sin[Q/2]) + \frac{K \cos[Q/2] \sin[K/2] - Q \cos[K/2] \sin[Q/2]}{K^2 - Q^2} \right) , \tag{8}$$

where natural units have been used for $K = \kappa d$ and $\Gamma = \gamma d$. Fig.5 and Fig.6 show the normalized far-field intensity distribution $|\tilde{\psi}|^2$ as a function of $Q = qd$ (natural units) and q (nm^{-1}) , respectively, to illustrate the functional changes with the parameter V. The width of the central peak defines the NA of the illumination. Aside from NA, the tails and the zeros also change with the parameter V. The blue curve ($V = \pi$) corresponds to the case where the fibre becomes mono-modal, i.e. to the cut-off potential V_{sm} below which only the fundamental mode can propagate. The orange curve corresponds to a stronger confinement ($V = 2\pi$), the green curve to a weaker confinement, where a higher fraction of the mode propagates in the cladding. Let´s briefly comment on the experimentally relevant curves shown in Fig.6, for an air/vacuum guide with Si cladding ($W = 19.9$nm). As d is reduced, the NA increases (from green to blue), but when $V < V_{sm}$ (orange), the mode can no longer be 'squeezed'. The confinement factor decreases and a higher proportion of the mode 'leaks' into the cladding. The loss of confinement results in a smaller NA but at the time the intensity tails extend to higher q, as the first zero moves in proportion to $1/d$. This could be exploited for holographic imaging without empty-beam division.

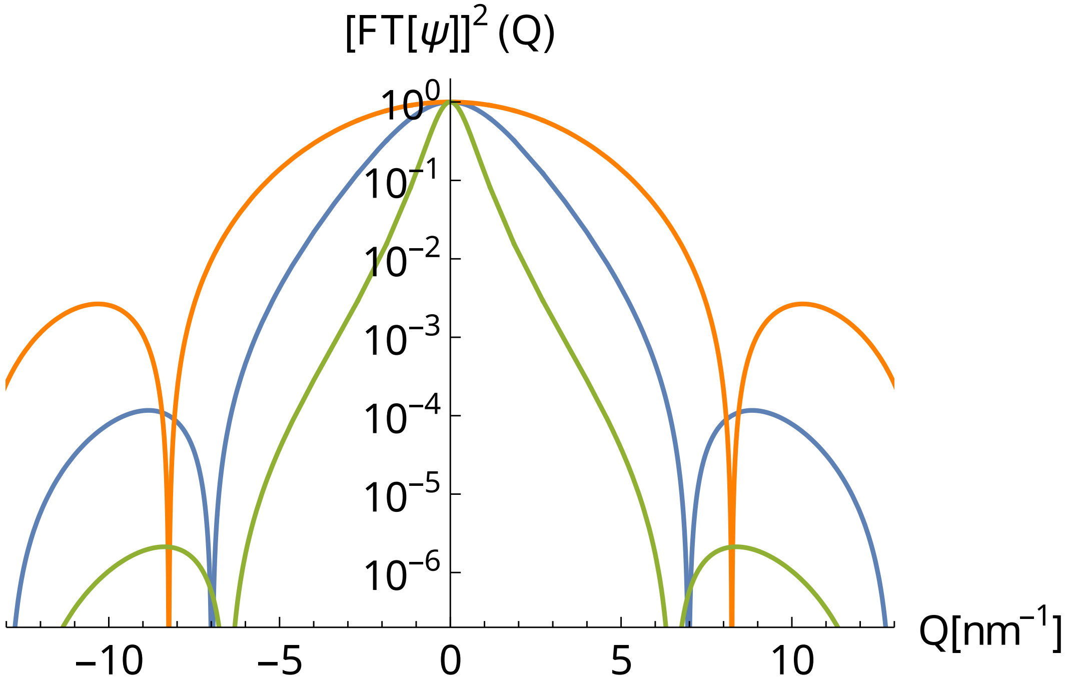

Figure 5: PWG/far-field. Far-field intensity of the fundamental mode in PWG model as a function of normalized lateral momentum transfer $Q = qa$, for different unitless potentials $V = \pi$ (blue), $V = 2\pi$ (orange), and $V = 0.44\pi$ (green). All curves have been normalized to the peak at $Q = 0$.

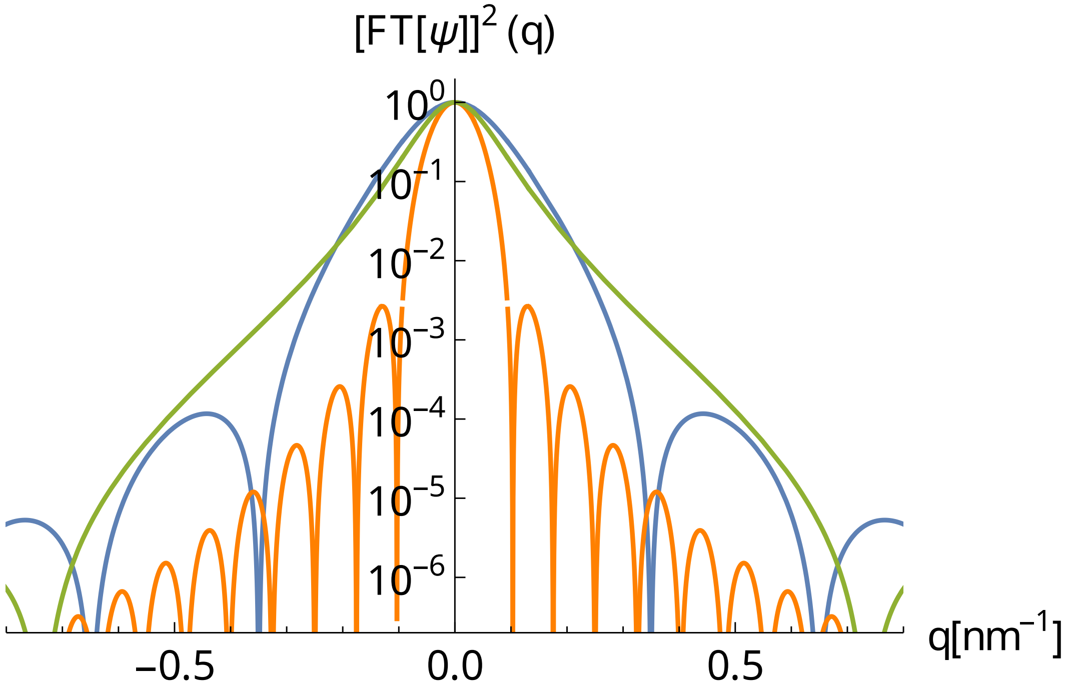

Figure 6: PWG/far-field. Far-field intensity of the fundamental mode in the PWG model (same as a above), but now as a function of lateral momentum transfer q, assuming that the PWG potential consists of vacuum/air core and Si cladding, corresponding to $W = 19.9$nm. Changes in V therefore relate to different d, notably to $d = 19.9$nm (blue), $d = 39.9$nm (orange), and $d = 8.8$nm (green), for $V = 1\pi$ (blue), $V = 2\pi$ (orange), and $V = 0.44\pi$ (green), respectively.

4.2 Weakly guiding fiber model (WGF)

As we saw, Ge surface diffusion during wafer bonding can result in cylindrical WG shape, motivating the use of weakly guiding fibers as an analytical model. The WGF model was introduced by Gloge (Gloge, 1971) for step-index fibers with a small difference between the index of refraction of core and cladding. It is an excellent approximation for the case of hard x-rays, and was discussed for this purpose already in (Fuhse, 2006). Let a denote the radius, r the radial and ϕ the azimuthal coordinate of the cylindrical system, and C_{lm} some normalisation constant. The linear polarized (LP) modes can be written as (Fuhse, 2006)

$$\psi_{\ell m}(r) = C_{\ell m} \begin{cases} \cos(\ell\varphi)\frac{J_\ell(u_m r/a)}{J_\ell(u_m)}, & \text{for } r < a, \\ \cos(\ell\varphi)\frac{K_\ell(w_m r/a)}{K_\ell(w_m)}, & \text{for } r \geq a. \end{cases} \tag{9}$$

The mode parameters u_m and w_m in core and cladding, respectively have to be calculated from a characteristic transcendental equation, and are linked by the relation $w_m = \sqrt{V^2 - u_m^2}$, where V is again the dimensionless potential of the waveguide (Marcuse, 1974; Gloge, 1971)

$$V := ka\sqrt{{n_1}^2 - {n_2}^2}. \tag{10}$$

If not mentioned otherwise, we consider the empty core WG with $n_1 = 1$ and $n_2 = 1 - \delta$, such that $V = ka\sqrt{2\delta} = ka\theta_c$. Switching to the notation of (Chen, 2006), we can also define a parameter b with $u_m = V\sqrt{1-b}$. For given V, the parameter b is then determined numerically from the equation

$$V\sqrt{1-b}\frac{J_1(V\sqrt{1-b})}{J_0(V\sqrt{1-b})} = V\sqrt{b}\frac{K_1(V\sqrt{b})}{K_0(V\sqrt{b})} \ . \tag{11}$$

For the lowest mode with $l = 0$ and no azimuthal variation $m = 0$, the solution reads

$$\psi_{00}(r) = C \begin{cases} \frac{J_0(V\sqrt{1-b}\ (r/a))}{J_0(V\sqrt{1-b})}, & \text{for } r < a, \\ \frac{K_0(V\sqrt{b}\ (r/a))}{K_0(V\sqrt{b})}, & \text{for } r \geq a\ . \end{cases} \tag{12}$$

This fundamental mode of these so-called LP-modes in plotted in Fig.7. The far-field distribution of the fundamental mode can be calculated by an analytical Fourier transformation (Gloge, 1971; Chen, 2006)

$$\tilde{\psi}(Q, V, b) := \frac{QJ_1(Q) - V\sqrt{1-b}\frac{J_1(V\sqrt{1-b})}{J_0(V\sqrt{1-b})}J_0(Q)}{(V^2(1-b) - Q^2)(V^2b + Q^2)} \ , \tag{13}$$

where J_n denotes the nth order Bessel function of the first kind. In Fig.8, the corresponding intensity is displayed $|\tilde{\psi}(Q)|^2$ is displayed for different V and b, computed for the fundamental model by solving the characteristic equation. In Fig.9, this result is translated into q in units of nm^{-1} for the specific choice of Ge and radius a = 5.4nm (orange), a = 10.9nm (blue), and a = 21.7nm (green). The latter is easiest to fabricate, with a diameter around 40 nm seeming

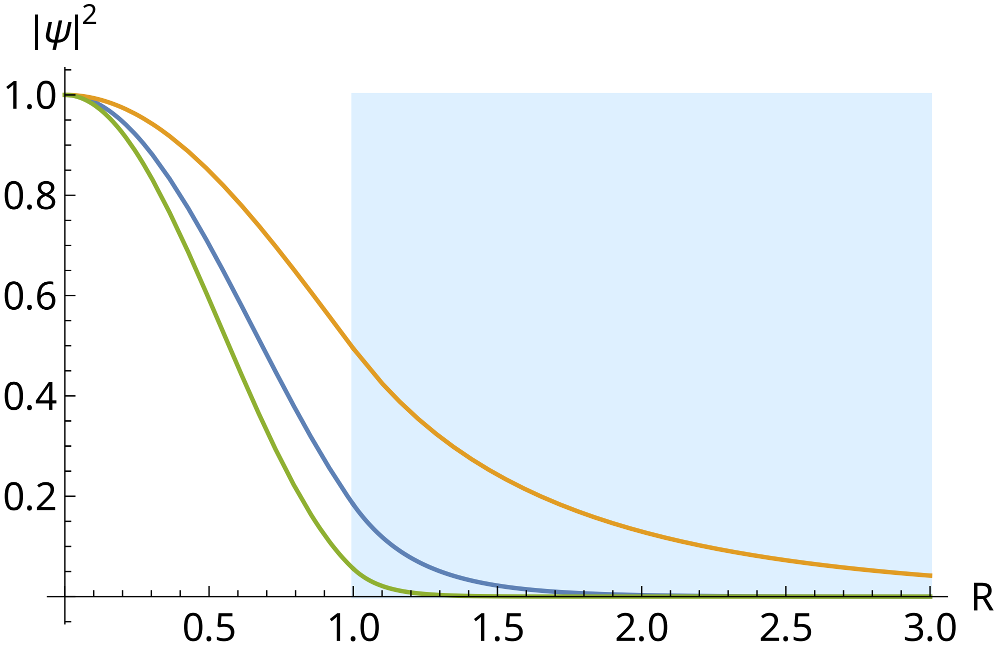

Figure 7: Normalized intensity of the fundamental LP mode in the WGF model as a function of radial distance $R = r/a$, for different unitless potentials $V = 1.2$ (orange), $V = 2.4048$ (blue), and $V = 4.8$ (green). The light blue region indicates the cladding. The blue curve ($V = 2.4048$) corresponds to the case where the fibre becomes mono-modal, i.e. at the cutoff point V_{sm} for the next higher mode (LP_{11}). For $V < V_{sm}$ only the fundamental mode can propagate as a guided mode. The fraction of intensity located in the core, the so-called confinement factor is 0.85 at the cutoff point, see the blue curve. Higher V (green) confine better, lower V results in pronounced tails residing in the cladding

a realistic value. Exploiting tails to $10^{-4} I(q = 0)$, one would reach $q \simeq 0.2nm^{-1}$. If diameters below $2a \simeq 20$nm could be fabricated, and with powerful pre-focusing optics to compensate the attenuation loss in the cladding, a smooth decay without oscillations could be designed, possibly reaching $q \simeq 0.4nm^{-1}$.

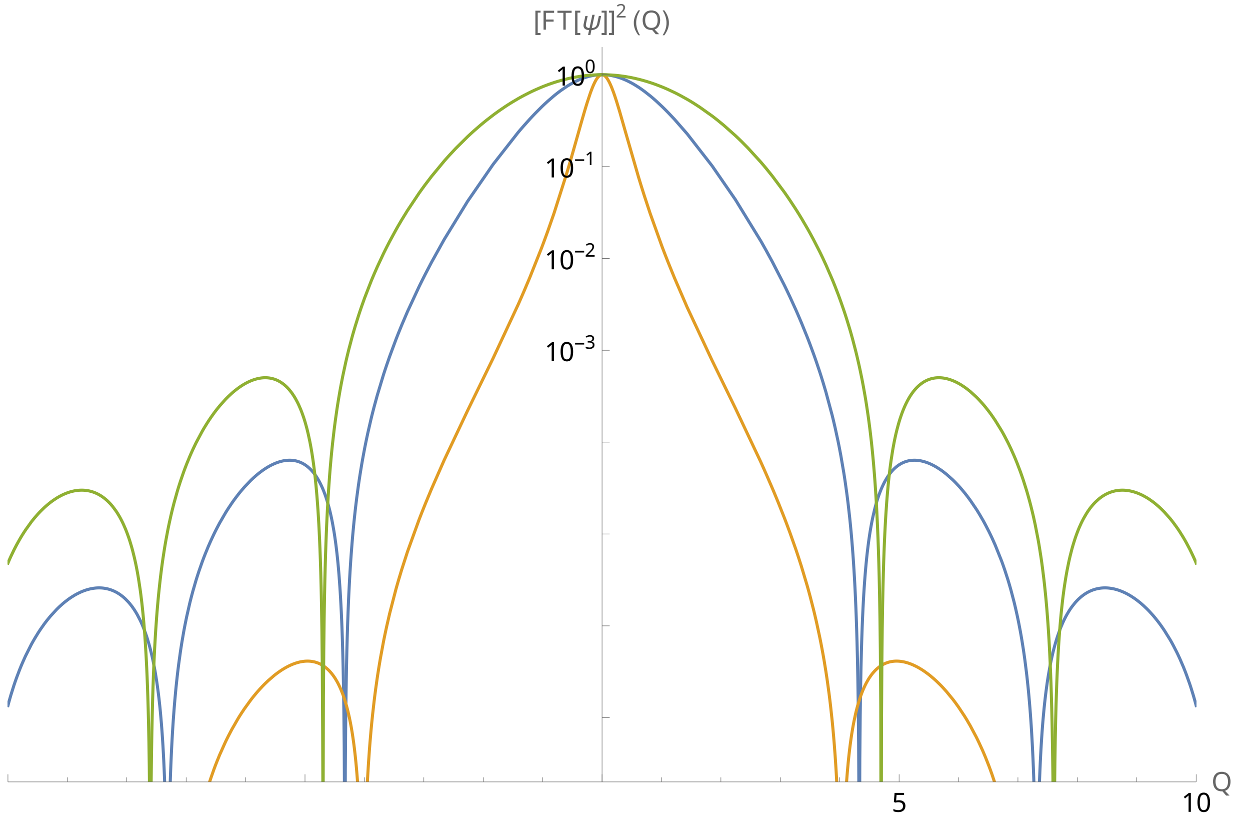

Figure 8: Far-field intensity of the fundamental mode in the weakly guiding fibre (WGF) model as a function of normalized lateral momentum transfer $Q = qa$, for different unitless potentials $V = 1.2$ (orange), $V = 2.4048$ (blue), $V = 4.8$ (green). All curves have been normalized to the peak at $Q = 0$, in order to illustrate the functional changes with the parameter V. The width of the central peak defines the NA of the illumination. Aside from NA, the tails and the zeros also change with the parameter V. The blue curve ($V = 2.4048$) corresponds to the case where the fibre becomes mono-modal, i.e. to the cut-off potential V_{sm} below which only the fundamental mode can propagate. The orange curve corresponds to a weaker confinement ($V = 1.2$), where a higher fraction of the mode propagates in the cladding.

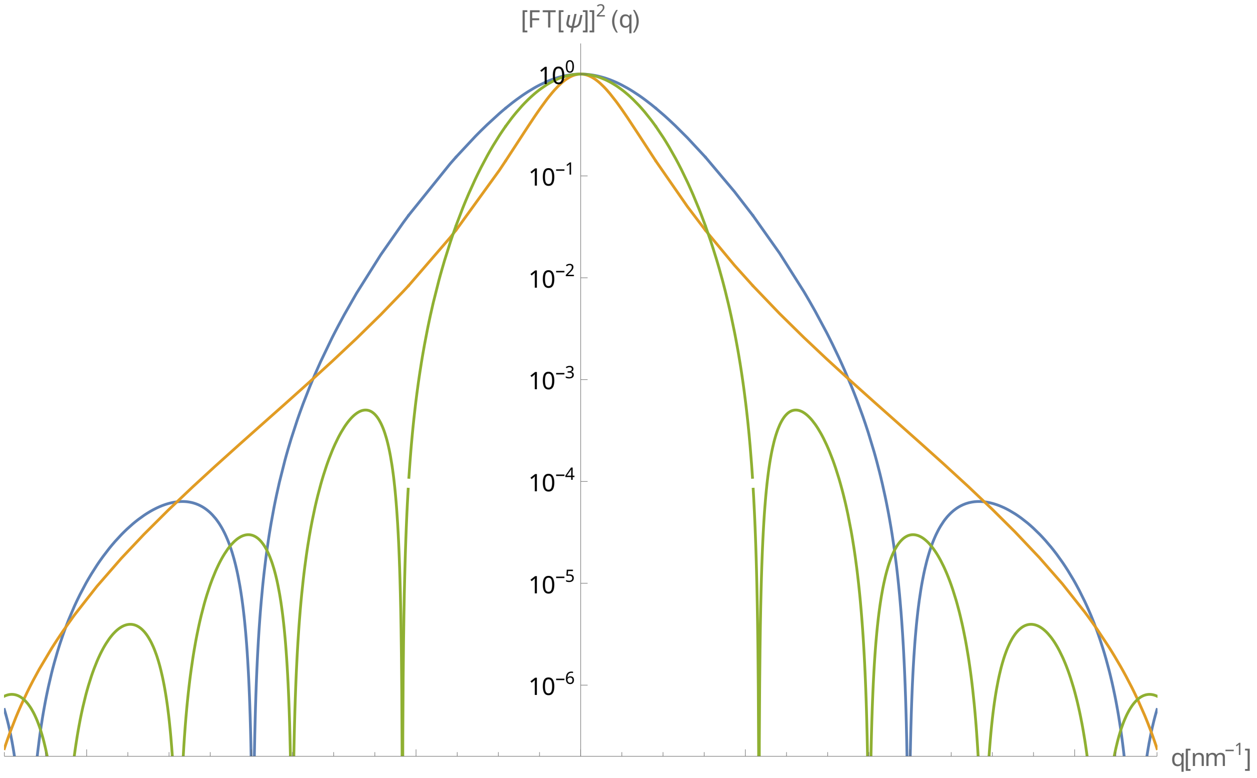

Figure 9: Far-field intensity of the fundamental mode in the WGF model (same as a above), but now as a function of lateral momentum transfer q, assuming that the WGF potential consists of vacuum/air core and Ge cladding, corresponding to $W = 14.2$nm. Changes in V therefore relate to different a, notably to $a = 5.4$nm (orange), $a = 10.9$nm (blue), and $a = 21.7$nm (green), for $V = 1.2$ (orange), $V = 2.4048$ (blue), $V = 4.8$ (green), respectively. Again, all curves are shown normalized to the peak at $q = 0$. As a is reduced, the NA increases (from green to blue), but when reducing to $V < V_{sm}$ (orange), the mode can no longer be 'squeezed'. The confinement factor decreases and a higher proportion of the mode 'leaks' into the cladding. The loss of confinement results in a smaller NA but at the time the intensity tails extend to higher q, as the first zero moves in proportion to $1/a$. This could be exploited for holographic imaging without empty-beam division.

5 Energy-independence of waveguide mode

Here we briefly recall the fact that away from absorption edges, x-ray waveguide modes are independent of photon energy (Salditt & Osterhoff, 2020). We rewrite the eigenvalue problem for planar waveguides as

$$\left[\partial_z^2 + k^2 n^2(z)\right] u(z) = k^2 \nu^2 u(z), \tag{14}$$

where ν is the effective refractive index (written as the propagation constant $\beta = k\nu$ in Eq.(1) of the main manuscript). We assume a 3-layer waveguide with refractive index profile

$$n(z) = \begin{cases} n_1 = 1 - \delta_1 & \text{for } |z| < L/2 \\ n_2 = 1 - \delta_2 & \text{otherwise,} \end{cases} \tag{15}$$

where $\delta_1 < \delta_2$. In particular, we neglect absorption. Substracting $k^2 n_1^2 u$ from both sides of (14), we obtain

$$\left[partial_z^2 - U(z)\right] u(z) = -\eta^2 u(z), \tag{16}$$

where $\eta^2 = -k^2(\nu^2 - n_1^2)$ and

$$U(z) = \begin{cases} 0 & \text{for } |z| < L/2 \\ U_0 := k^2(n_1^2 - n_2^2) & \text{otherwise.} \end{cases} \tag{17}$$

Equation (16) is just the well-known eigenvalue problem of the finite potential well. Next we insert the x-ray refractive index. Neglecting resonant scattering (near the absorption edges), Thomson scattering results in a direct proportionality between the decrement δ and the electron density ρ_e

$$n = 1 - \delta \tag{18}$$

$$\delta = \frac{2\pi r_0 \rho_\mathrm{e}}{k^2}, \tag{19}$$

so that the potential becomes

$$\begin{aligned} E_0 &= k^2(n_1^2 - n_2^2) \approx 2k^2(\delta_2 - \delta_1) \\ &= 4\pi r_0(\rho_{\mathrm{e},2} - \rho_{\mathrm{e},1}), \end{aligned} \tag{20}$$

which is hence independent of wavelength / photon energy E.

6 Coherence

Statistical optics in general, and speckle visibility and intensity probability distribution in particular offer a suitable way to assess coherence properties, even without involved interferometric instrumentation or multi-acquisition Talbot scans (Salditt *et al.*, 2011). As a control of nearly full spatial coherence, we here test the intensity probability distribution function (pdf) of holograms recorded behind a statistical pseudo-random diffuser, notably a random hole diffuser (RHD), made of 200nm wide holes positioned at pseudo-random locations in a 1.5μm thick layer of W with, deposited on a thin silicon nitride window (Zonplate.com Ltd, UK). A similar structure was used in (Lee *et al.*, 2023), for optical different purposes. The total covered by holes was 22%. An overall transmission $T = 0.86$ of the structure was measure at $E = 13.8$keV. Different realisations of

pattern were available on the same foil, arranged in a 4×4 array, each within a circular region of 80μm diameter. The diffuser was illuminated by the $Ru/B_4C/Ru$ WGx-type WG ($d = 50$nm, L=2mm, $Ru/B_4C/Ru$) at at $E = 13.8$keV. Ptychographic scans were acquired at a defocus distance $z_{01} = 2$mm and magnification $M = 2550$, each scan position illuminating a field of view (FOV) of about 10μm, within one circular region of the RHD.

Fig. 10 presents statistical analysis of the near-field speckle measured behind the RHD. In (a), the empty-beam normalized hologram in the center of the waveguide beam (150×150 pixel wide cutout) is shown, and in (b) the corresponding pdf, along with a one-parameter least-square fit to the model of a constant phasor plus a random phasor sum (solid line) (Goodman, 2020). The high visibility and abundant occurrence of nearly perfect destructive interference can be well evidenced based on the essentially background-free photon-counting capability of the Eiger 4M pixel detector (Dectris, Baden, Switzerland). For the phase of a far-field speckle pattern, an exponential pdf indicates full coherence, while the presence of M spatial modes reduces the occurrence of perfect destructive interference, and results in a Γ-function pdf. For an application to coherence assessment of a free electron laser beam, see for example (Mai *et al.*, 2013). In the near-field case, the situation is different, as the scattered waves of the modulator interfere with the reference wave, i .e. the speckle pattern is formed by a sum of a single 'constant' or known phasor, plus a random phasor sum with a uniformly distributed phase (Goodman, 2020). Denoting the reference intensity by I_0 and the random phase sum as I_n with mean $\hat{I}_n$, the following pdf can be derived in this model under the assumption of full coherence

$$p(I) = \frac{1}{\bar{I}_n} \exp[-\frac{I + I_0}{\bar{I}_n}] \; Bessel I_0 \left(2 \frac{\sqrt{I I_0}}{\bar{I}_n} \right) \; . \qquad (21)$$

Note that in this model, the only open parameter is the ratio of I_0 and $\hat{I}_n$, since $I_0 + \hat{I}_n$ is constrained by the measured integrated intensity. When the pdf is normalized to its mean, we therefore have $I_0 + \hat{I}_n = 1$. The least-square fit to the resulting one-parameter curve fits the data well for a relative contribution of $I_0 = 0.63$ and $\hat{I}_n = 0.37$. Note that this value depends on the Fresnel number of data acquisition, since the relative weight of diffracted contributions increases with effective propagation distance, relative to the primary reference beam. In other words, in self-interference becomes stronger. While potentially much more information can be deduced from the near-field speckle analysis, we can leave it at this for the present proof of nearly full-field spatial coherence of the waveguide beam.

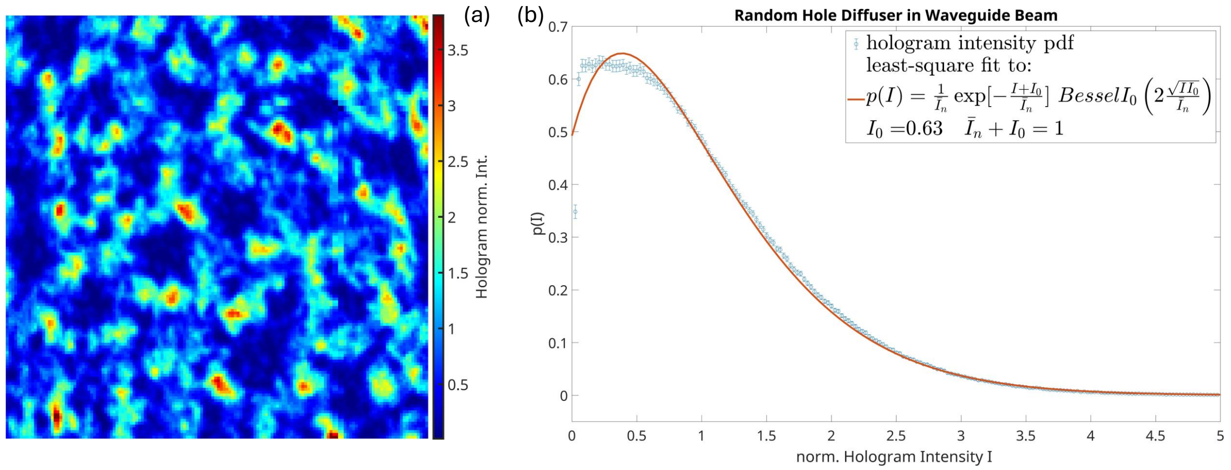

Figure 10: Near-field speckle for coherence analysis. The hologram intensity distribution is measured behind a random hole diffuser (RHD) used as a statistical wavefront modulator in the waveguide beam. (a) High visibility of the of the hologram exhibiting a pronounced statistical distribution of positive and negative interference behind the RHD screen, created by the pseudo-random positioning of 200nm holes in the 1.5μm W mask. The image shows a 150×150 pixel wide cutout of the central waveguide beam, recoded with the photon counting Eiger 4M pixel detector (Dectris, Baden, Switzerland) . (b) Intensity probability distribution function (pdf) computed after normalisation to the mean intensity, averaged over 121 patterns from a ptychographic scan at $z_{02} = 2$mm behind the waveguide exit. The pdf is well accounted for by the statistical model of a constant phasor plus a random phasor sum (one-parameter least-square fit). This model assumes full coherence, and the good agreement between model and data, in particular the pronounced presence of negative interference down to zero value.

References

Chen, C.-L. (2006). *Foundations for guided-wave optics.* John Wiley & Sons.

Fuhse, C. (2006). *X-ray waveguides and waveguide-based lensless imaging.* Ph.D. thesis, University of Göttingen.

Gloge, D. (1971). *Applied optics,* **10**(10), 2252–2258.

Goodman, J. W. (2020). *Speckle phenomena in optics.* SPIE.

Hoffmann-Urlaub, S. (2016). *X-ray waveguide optics: Beyond straight channels.* Ph.D. thesis, Universität Göttingen.

Hoffmann-Urlaub, S., Höhne, P., Kanbach, M. & Salditt, T. (2016). *Microelectron. Eng.* **164**, 135–138.

Hoffmann-Urlaub, S. & Salditt, T. (2016). *Acta Crystallogr. A,* **72**(5), 515–522.

Lee, K., Lim, J., Lee, S. Y. & Park, Y. (2023). *Light: Science & Applications,* **12**(1), 88.

Mai, D. D., Hallmann, J., Reusch, T., Osterhoff, M., Düsterer, S., Treusch, R., Singer, A., Beckers, M., Gorniak, T. & Senkbeil, T. (2013). *Opt. Express,* **21**(11), 13005–13017.

Marcuse, D. (1974). *Theory of Dielectric Optical Waveguides.* Acad. Press, New York.

Osterhoff, M. (2012). *Wave optical simulations of x-ray nano-focusing optics.* Ph.D. thesis, Universität Göttingen.

Osterhoff, M. & Salditt, T. (2009). *Optics Communications,* **282**(16), 3250–3256.

Salditt, T., Kalbfleisch, S., Osterhoff, M., Krüger, S. P., Bartels, M., Giewekemeyer, K., Neubauer, H. & Sprung, M. (2011). *Opt. Express*, **19**(10), 9656–9675. http://www.opticsexpress.org/abstract.cfm?URI=oe-19-10-9656

Salditt, T. & Osterhoff, M. (2020). *Nanoscale Photonic Imaging: X-ray Focusing and Optics*, pp. 35–70. Springer International Publishing. https://doi.org/10.1007/978-3-030-34413-9-3

Soltau, J., Vassholz, M., Osterhoff, M. & Salditt, T. (2021). *Optica*, **8**(6), 818–823.
